# Modeling Neoplastic Growth in Renal Cell Carcinoma and Polycystic Kidney Disease

**DOI:** 10.3390/ijms22083918

**Published:** 2021-04-10

**Authors:** Cassandra Millet-Boureima, Stephanie He, Thi Bich Uyen Le, Chiara Gamberi

**Affiliations:** 1 Department of Biology, Concordia University, Montreal, QC H4B 1R6, Canada; cassandra.millet@mail.concordia.ca (C.M.-B.); stephanie.he@concordia.ca (S.H.); Thi_Bich_Uyen_LE@nuhs.edu.sg (T.B.U.L.); 2 Haematology-Oncology Research Group, National University Cancer Institute, Singapore 119228, Singapore; 3 Department of Biology, Coastal Carolina University, Conway, SC 29528-6054, USA

**Keywords:** renal cell carcinoma, polycystic kidney disease, *Drosophila*, disease model, neoplasia, neovascularization, cilia, oxygen, pharmacology

## Abstract

Renal cell carcinoma (RCC) and autosomal dominant polycystic kidney disease (ADPKD) share several characteristics, including neoplastic cell growth, kidney cysts, and limited therapeutics. As well, both exhibit impaired vasculature and compensatory VEGF activation of angiogenesis. The PI3K/AKT/mTOR and Ras/Raf/ERK pathways play important roles in regulating cystic and tumor cell proliferation and growth. Both RCC and ADPKD result in hypoxia, where HIF-α signaling is activated in response to oxygen deprivation. Primary cilia and altered cell metabolism may play a role in disease progression. Non-coding RNAs may regulate RCC carcinogenesis and ADPKD through their varied effects. *Drosophila* exhibits remarkable conservation of the pathways involved in RCC and ADPKD. Here, we review the progress towards understanding disease mechanisms, partially overlapping cellular and molecular dysfunctions in RCC and ADPKD and reflect on the potential for the agile *Drosophila* genetic model to accelerate discovery science, address unresolved mechanistic aspects of these diseases, and perform rapid pharmacological screens.

## 1. Introduction

Abnormal neoplastic growth underlies benign and malignant neoplasms or tumors. While benign tumors may grow slowly and do not spread to other sites, malignant cancerous growth displays variable propensity to form metastases to other tissues. Neoplastic cells are characterized by variably altered cellular and energy metabolism, high mutation rates, and genomic instability (reviewed in [1]). In typically benign cysts, neoplastic growth creates a sac filled with liquid. Cystic growth may precede carcinogenesis, and several cancers can form cysts, albeit the relationship between cysts and cancer and possible causality remain unresolved. Among renal neoplasias, renal cell carcinoma (RCC) and autosomal dominant polycystic kidney disease (ADPKD) affect the renal tubules, share several characteristics, and appear both distinct and interrelated. Our knowledge of the precise molecular mechanisms of both RCC and ADPKD is incomplete, which limits progress toward effective therapeutics. Thus, genetic models of neoplastic growth in RCC and ADPKD are expected to help advance our understanding of the underlying molecular lesions and their possible cooperation in disease etiology. Here, we examine the current knowledge of RCC and ADPKD, with particular interest in the molecular lesions underpinning the abnormal cell growth and discuss the potential to use the genetic tools in *Drosophila* to address unresolved mechanistic aspects and to accelerate drug discovery and testing.

The most common form of kidney cancer found in adults, largely sporadic RCC affects 1 to 15:100,000 people depending on the geographical location, with the highest rates in the Czech Republic and North America [2,3]. RCC accounts for 90% of all adult kidney malignant tumors and 10% of all cancers [4,5,6]. It is also twice more frequent in men than women [7]. RCC is the 13th most common cause of cancer mortality worldwide [8], and it consists of a group of related malignancies sharing similar histology, common anatomical origin in the nephron, diverse molecular signatures, and therapeutic outcomes ([9,10], reviewed in [11]). RCC presents three major subtypes: clear cell RCC (ccRCC), papillary RCC (pRCC) and chromophobe RCC (chRCC), as well as less frequent subtypes [12] that have been linked to several genetic mutations (Table 1) ([13,14]. RCC subtypes have been reviewed recently [15]. This review will focus on ccRCC. Tumors in ccRCC are commonly classified using the Fuhrman nuclear grade that consists of diagnostic traits to identify cancer progression from early G1 stage with barely abnormal-looking cells to advanced G4 cancers with numerous often grotesque cellular abnormalities (Table 2). Thirty-five percent of RCC patients develop bone metastases, especially in the ribs, pelvis, and/or spine [16], which are important prognostic determinants. RCC has a 40% fatality rate [17,18,19].

ADPKD is hereditary and affects 1:500 to 1:800 people worldwide regardless of ethnicity (reviewed in [34]). ADPKD causes the formation of fluid-filled cysts in the nephron [34]. Already visible at birth, ADPKD-type cysts enlarge over time as new cysts begin to form, progressively distorting and compressing the surrounding parenchyma and causing fibrosis and end-stage renal disease in about half of the patients [34]. The precise molecular underpinning of ADPKD is largely unresolved. However, multiple cellular and physiological functions including cell proliferation and apoptosis are known to be disrupted, and cystic growth presents some neoplastic characters. In addition to contributing to cystic growth, ADPKD upsets polarization of the tubular epithelium. Increased fluid secretion in early disease stage triggers compensatory vasopressin release and systemic responses affecting the cardiovascular system (reviewed in [35]). Moreover, ADPKD patients suffer from several extra-renal manifestations (reviewed in [34]). Over 90% of ADPKD cases have been linked to mutations in either the *PKD1* or *PKD2* genes, with the former being defective in ~85% of the cases, the latter in 10–12% of the patients, and the remaining 3–5% being due to other mutations [34]. The *PKD1* and *PKD2* cognate proteins, called polycystin 1 (PC1) and polycystin 2 (PC2), are widely distributed in the cell. Part of the cellular PC1 and PC2 pools form complexes with one another [36]. The wide cellular distribution of the PC1-PC2 complexes and their multifaceted involvement in organellar function e.g., non-motile cilia, yield functional complexity the detail of which is challenging to discern experimentally [37].

RCC is often associated with cysts. Cysts are found in the infrequent cystic RCC and the prevalent ccRCC and pRCC (Table 1) [38,39,40]. Cysts may prelude RCC [40,41], however, important aspects such as causality and the extent of cyst heterogeneity remain to be determined.

## 2. Neoplastic Character and Vasculature

**Figure 1:**
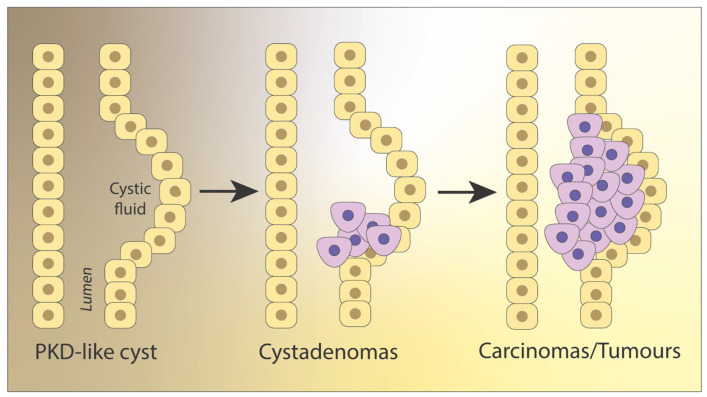
RCC progression. In many cases, clear cell renal cell carcinoma (ccRCC) begins as PKD-like cysts in the renal tubules that progress into cystadenomas, and malignant and invasive tumors. Adapted from [46].

Although no direct link has been specified between ADPKD and predisposition to develop RCC, J. Grantham has inferred that the late stage of kidney disease (also known as acquired cystic kidney disease, ACKD) could be seen as “neoplasia in disguise”, due to abnormally growing epithelial cells at the side wall region that resemble multifocal neoplasia [42,43]. Note, unlike having multiple tumors, multifocal neoplasia has been described as originating from a unique cell clone, which then grows multifocally in a single organ [44]. ACKD commonly precedes multifocal renal adenoma and frank adenocarcinoma [42]. Among neoplasias, cysts are defined as abnormal membranous sacs or cavities containing fluid, adenomas as benign tumors derived from glandular structures in epithelial tissue, and tumors as benign or malignant swellings caused by abnormal tissue growth. Differentiating between cysts, adenomas, and tumors may be complicated by the presence of partially overlapping morphologic and molecular features yet is expected to empower prognosis. Morphologically, cysts and adenomas similarly display enlarged epithelial walls, where the adjacent parenchyma is compressed and collapses [43]. However, ADPKD cysts are filled with fluid from glomerular filtrate, whereas adenomas are packed with cells [43]. Suggesting commonalities, ccRCC may begin as ADPKD-like cysts in the nephron, which transform into cystadenomas, and eventually lead to malignant and invasive tumors (Figure 1) [45,46].

Molecularly, ADPKD cysts and ccRCC share similarities. Conditional deletion of the *Vhl* and *Pbrm1* genes (also implicated in ccRCC) in the cells of the murine renal epithelium may cause preneoplastic PKD and eventually ccRCC, albeit the single mutations do not [45]. A widely utilized murine model of RCC, *Vhl*^F/F^*Pbrm1*^F/F^*Ksp-Cre* mice were generated by Cre/lox recombination in the Ksp-Cre line, that harbors the Ksp-cadherin kidney-specific promoter [45,47]. The *Vhl*^F/F^*Pbrm1*^F/F^*Ksp-Cre* mice tend to develop multifocal, clear cell kidney cancer with a 50% tumor incidence after ten months of age [45]. Moreover, such mice showed characteristics similar to VHL patients, i.e., a significantly higher mortality rate, elevated serum creatinine and occurrence of preneoplastic PKD-type cysts in both tubules and glomeruli by six months of age [38,45,48].

Vasculature appears abnormal in ADPKD, autosomal recessive (AR)PKD (reviewed in [49]), and cancer [50,51,52]. The peritubular reticular capillaries of the renal cortex regulate fluid balance through reabsorption of the glomerular filtrate. In ADPKD, the renal blood vessels are remodeled, which is thought to contribute to disease progression [49,53]. In both ADPKD patients and the *Pkd1^nl/nl^* mice, the peritubular capillaries become spiral-shaped, convoluted, and dilated [54,55,56]. Compared to wild type, the *Pkd1^nl/nl^* mice also have fewer vessel segments and branches [57]. The lymphatic capillaries that normally clear the interstitial fluid from around the organs are also remodeled, exhibit several malformations and decreased branching [58]. Compromised lymphatic function causes blood accumulation in the lymph sacs and contributes to early lethality of the *Pkd1^−/−^*, *Pkd1^nl/nl^*, *Pkd1^RC/RC^*, and *Pkd2^−/−^* mice [49,57,59,60]. Reminiscent of tumor growth, cyst expansion also requires a shift in the vasculature to provide metabolites to support angiogenesis, through a temporary repair mechanism generating new vessels from existing vasculature to supply blood to the repaired tissue [61]. A primary growth factor promoting angiogenesis is vascular endothelial growth factor (VEGF)-A, which binds to receptor VEGFR-2 to promote blood endothelial cell proliferation, differentiation, migration, and survival [62]. Expressed in cortical tubules and large cysts, VEGF-A could promote cyst expansion through increased angiogenesis [49,63]. In lymphatic endothelial cells, activation of another growth factor, VEGF-C, and its receptor VEGFR-3, also promotes cell proliferation, differentiation, migration, and survival [60], but the specifics are not fully understood [49]. Current knowledge suggests that VEGF-C activation may ameliorate PKD by increasing lymphatic vessels, leading to the clearance of excess fluids and inflammatory cells from the renal interstitial space [56].

VEGF drives sustained angiogenesis in cancer, yielding abnormal, overly permeable, and disorganized blood vessels [50,51,52]. In particular, ccRCC exhibits complex neovascularization in which VEGF upregulation drives abnormal increase of capsular vascular supply with outflow through the ovarian or testicular veins [64,65]. Despite such increased supply, blood perfusion in the tumor itself appears less than in normal renal tissues [65,66], possibly because ccRCC typically builds a network of small sinusoidal blood vessels [67]. Understanding how the complex vasculature and lymphatic system function and remodel is expected to yield novel biomarkers and therapeutic targets for ccRCC [67] and, possibly, for ADPKD.

## 3. Molecular Pathways and Genes Implicated in RCC

**Figure 2:**
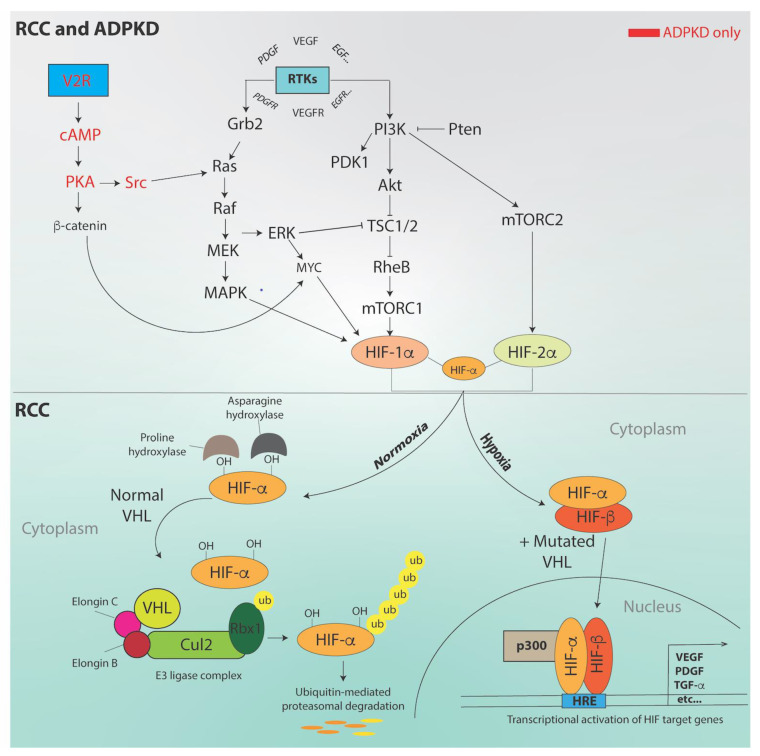
Signal transduction in RCC and autosomal dominant polycystic kidney disease (ADPKD) progression. See text for details. Adapted from [84,85].

Indicative of genetic instability, RCC tissues display numerous chromosomal aberrations, especially in advanced stages [68,69,70,71,72,73,74,75,76,77]. Loss of the short arm of chromosome 3 during early life removes one copy of the *von Hippel-Lindau* (*VHL*) gene, which is considered the “first hit” predisposing to ccRCC [11]. The *VHL* gene encodes a protein that normally interacts with proteins Elongin B and C, Cul2, and Rbx1 to form a ubiquitin E3 ligase complex [78]. Under normoxia, hypoxia-inducible-factor-(HIF)-α gets hydroxylated by prolyl hydroxylase domain (PHD) proteins, which increases its affinity for VHL (Figure 2) [79]. Two HIF isoforms, HIF-1α and HIF-2α, are found respectively in tubular epithelial cells and the glomerular and peritubular cells, where erythropoietin (EPO) is produced [79,80]. Normally, the VHL-containing ubiquitin E3 ligase complex targets HIF-1α for proteasomal degradation (Figure 2) [79,81]. During hypoxic stress, HIF-1α and HIF-2α are stabilized and translocated to the nucleus to form a heterodimer with a stable and constitutively expressed HIF-1β subunit [79]. Transcriptional coactivators including p300/CBP, along with the HIF heterodimers, bind to hypoxia response elements (HREs) to induce transcription of hypoxia target genes (e.g., *VEGF, glucose-transporter (GLUT1), EPO*) and initiate the cell response to oxygen deprivation (Figure 2) [79,82]. Also involved in the hypoxic response and promoting vascularization in neighboring endothelial cells, the *epidermal growth factor receptor* (*EGFR*) and *platelet-derived growth factor* (*PDGF*) genes do not contain HREs and appear subject to different regulatory cues [82,83]. Together, these factors are thought to support energy metabolism during carcinogenesis.

A “second hit” that eventually leads to tumorigenesis is caused by somatic mutations or epigenetic modifications inactivating the second copy of *VHL* [86,87]. Frequently, additional mutations are found in ccRCC, often in distinct clones of cells within the same tumor (Table 1) ([88,89], reviewed in [11]). These hits include genes *Polybromo-1* (*PBRM1*), *BRCA1-associated protein 1* (*BAP1*), *SET domain-containing 2* (*SETD2*), *Lysine demethylase* (*KDM5C*), *mTOR*, *phosphatase and tensin homolog* (*PTEN*), *PI3K catalytic subunit* (*PIK3CA*), *tumor protein P53* (*TP53*), and *Elongation Factor B* (*TCEB1*) [11,90,91,92,93].

Murine ccRCC models and human ccRCC cancers feature mTOR activation and cellular overgrowth after loss of *VHL* and *PBRM1* gene function (Figure 2) [45]. However, the mouse and human genomes, normally largely syntenic, display different arrangement of the homologous RCC genes [11]. Therefore, mice models cannot faithfully reproduce the genomic rearrangements leading to human ccRCC. Other contributors to the development of ccRCC include key signal transduction pathway PI3K/Akt/mTOR, HIF-1α, HIF-2α, VEGF, EGFR, carbonic anhydrase-IX (CA-IX), GLUT transporters, transforming growth factor-(TGF)-α, TGF-β, and Notch [94,95]. Furthermore, the VHL/HIF and PI3K/AKT pathways have shown to cross-talk in an extensive signaling network contributing to ccRCC progression [96]. These cascades affect tumor cell formation, cell fitness, angiogenesis, and migration.

mTOR can be activated in response to autocrine and paracrine growth factors (e.g., EGF-1, IGF-1, PDGF, TGF-α), that lead to receptor tyrosine kinase transphosphorylation, upregulation of PI3K/AKT/mTOR, Ras/Raf/ERK, and MAPK that phosphorylate multiple cytoplasmic components. Further augmenting mTOR activity, AKT and ERK downregulate mTOR modulators such as PTEN and tuberous sclerosis complex (TSC1/2). Activated AKT inhibits apoptosis by phosphorylating the proapoptotic procaspase-9 and Bcl2 proteins and by activating proliferation-promoting transcription factors such as β-catenin, c-Jun, c-Myc, and Notch [97]. mTOR also drives components of the translational machinery S6K, 4EBP-1, and sterol regulatory element-binding protein (SERBP)-1, that support cell proliferation and lipid metabolism in tumor cells [98,99]. All the pathways mentioned above are also found dysregulated in ADPKD (see Figure 2, reviewed in [100]). In ADPKD, PKA abnormally activates the Src/Ras/Raf/MEK/ERK pathway, that is instead inhibited in wild-type renal cells [101]. The mTOR pathway is also hyperactive in ADPKD, supporting cystic cell growth and proliferation [102,103,104]. In ADPKD kidneys, under normoxic and hypoxic conditions, PI3K/mTOR signaling activates HIF-1α expression, which leads to autophagy [105,106]. Concurrent c-Myc upregulation promotes cell proliferation and, together with increased Bcl-2 expression and reduced p53 protein levels, also dysregulates apoptosis [107,108]. The V2R signaling cascade becomes progressively upregulated in ADPKD, which raises intracellular cAMP levels and characteristically contributes to cystic cell growth and proliferation (reviewed in [35]). Of note, in normal renal epithelial cells, high cAMP levels inhibit cell proliferation (reviewed in [109]).

## 4. Cell Metabolism

Cancer is known to alter cell metabolism and respiration to suit tumor cell growth, a phenomenon called the Warburg effect [95]. ccRCC has been considered a type of metabolic disease on the basis of the upregulation of several gene products e.g., VHL, MET, fumarate hydratase (FH), folliculin (FLCN), succinate dehydrogenase (SDH) and, TSC1/2 that regulate the mTOR pathway, PTEN, and other energy metabolism-related pathways [110]. A classic example of the Warburg effect, SDH and FH deficient kidney cancers undergo a metabolic shift to perform aerobic glycolysis, and boost production of ATP and metabolites needed for rapid cell growth and division [110,111].

Glucose metabolism appears to fuel several types of rapidly growing tumor cells [112]. Increased glycolysis and glucose uptake in malignant cells and higher tumor grades are supported by higher expression of the GLUT transporters, promoted by oncogenes and growth factors [113]. Also regulating metabolism in ccRCC is CA-IX, a tumor-associated glycoprotein induced by hypoxia which is involved in cancer progression [114]. CA-IX regulates the intracellular pH and generates a surplus of acidic products, leading to cancer cell survival and proliferation [114]. Moreover, CA-IX was found to be a HIF-1α target in ccRCC [115]. Corroborating the view of commonalities between (at least some) cysts and (at least some) cancer, murine models of papillary type II carcinoma reproduced a gradual transformation from benign cysts into cystadenomas and carcinomas, mediated by fumarate-driven epithelial to mesenchymal transition and the mTORC1 cascade [46,116]. Two RCC patients also displayed a gradual transformation from benign cysts to neoplasia, suggesting that simple benign renal cysts may transform into RCC and underscoring need for a prudent practice of regular cyst monitoring [117]. Similar mTOR-driven metabolic regulation was found in another RCC type in a kidney-specific Cre (KspCre) *Tsc1* mutant restricted to a segment of renal tubule; in this case, mTOR has been found to downregulate the TCA cycle enzyme fumarate hydratase leading to fumarate accumulation, and driving tumor cell progression [46,116]. These results suggest that the mTOR-FH axis is critical to oncometabolite production, and crucial metabolic machinery may be critical to stabilizing cancer cell fitness.

ADPKD has recently been reported as displaying mitochondrial dysfunction similar to metabolic disease [79]. Reminiscent of cancer cells [118], the Warburg effect has been observed in *Pkd1^−/−^* mouse embryonic fibroblasts, that were dependent on aerobic glycolysis to produce the energy needed to fuel cellular growth and proliferation [119]. Compared to *Pkd1^+/+^* controls, *Pkd1^−/−^* mice, along with microarrays from ADPKD patients, revealed enhanced glycolysis [119]. *Pkd1^−/−^* cells appeared to consume more glucose, produce more lactate and have increased ATP content, leading to glucose deprivation [119]. When glucose levels were reduced, the proliferation of cystic cells was lowered to wild-type level [119]. Glucose deprivation also increased apoptosis and abnormal autophagy in *Pkd1^−/−^* cystic mice, whereas *Pkd1^+/+^* cells activated autophagy to survive [119]. Treatment with glucose analog and competitive glucoisomerase inhibitor 2-deoxyglucose, effectively targeted glycolysis, and reduced the cystic index (cyst number) without any effect on other organs and body weight [119]. Consistent with these observations, caloric restriction and ketosis slowed disease progression in the ADPKD mice [120,121].

Finally, HIF-1α appears to promote the Warburg effect and has been found upregulated in *Pkd1^−/−^* mouse embryonic fibroblasts compared to control *Pkd1^+/+^* cells [119]. In ADPKD, cystic epithelial cells overexpress GLUT1 [122], which is in turn under HIF-1α regulation [123]. Underscoring multiple levels of regulation, the *HIF-1α* mRNA is a target of mTOR-mediated translational control [105].

## 5. Oxygen in ADPKD

ADPKD features regional hypoxia and activation of the HIF pathway. During hypoxia, HIF-1α is upregulated in the cystic epithelial cells, whereas HIF-2α is expressed in pericystic stromal and endothelial cells [79,124]. Both HIF protein and mRNA amounts positively correlated with the cystic index in murine models [124,125] and ADPKD patients [126]. In ADPKD, HIF-1α does not affect early cyst formation but rather, it is important for cyst growth and enlargement in later stages of the disease [79]. In contrast, in RCC, HIF-1α expression remains stable during hypoxia [79]. Madin–Darby canine kidney (MDCK) cells that resemble principal cells ((pl)MDCK) are often used to model cyst formation in tissue culture. In MDCK cells, decreased oxygen concentrations correlated with increased cyst size [125]. Conversely, HIF-1α inhibition by chetomin reduced cystic growth [125]. In conclusion, HIF pathway activation during hypoxia is a novel mechanism contributing to cyst enlargement in ADPKD [79].

Independent of the HIF pathway, reactive oxygen species (ROS) have also been shown to promote cyst progression in ADPKD. ADPKD tissue displays increased ROS levels that are positively correlated with disease severity [79,127].

## 6. Ion Channel Signaling

Ion channels and ion pumps appear to contribute to tumor progression through the regulation of adhesion complexes and surrounding extracellular matrix (ECM) proteins that modulate cell motility, shape, and volume [128].

The cytoplasmic chloride ion channel protein-1 (CLIC1) is normally expressed in the glomerulus and epithelial cells of the kidney proximal tubules [129]. CLIC1 is overexpressed in many tumor types [130] and drives glioblastoma cell growth and proliferation [131]. In response to oxidative stimuli and malignant transformation, CLIC1 translocates from the cytoplasm to the cell membrane [132,133,134], where it functions as a selective chloride channel that affects cell cycle regulation, cell size, membrane potential, and cell proliferation/differentiation [130,135]. As expected, high CLIC1 expression has been found in 50 resected samples of human ccRCC in both malignant tissue and sites of intravascular invasion, with G3 tumors displaying the most heterogeneous CLIC1 distribution [130]. CLIC1 was found to be expressed in the cytoplasm, membrane, and nucleus of tumor cells, indicating that it could play several biological functions depending on location [130]. Although the CLIC1 role in ccRCC has not been fully elucidated, several primary cell lines from ccRCC patients indicated that CLIC1 inhibition blocked the myosin light chain kinase (MYLK) and β3 integrin, decreasing tumor proliferation and slowing cancer progression [136]. To date, there seems to be no evidence implicating CLIC1 in ADPKD.

In ADPKD, the CFTR protein acts as a cAMP-dependent chloride channel for Cl^-^ secretion and fluid production [137]. Normally, the CFTR mRNA is abundant in the nephrons, but not the glomeruli [137]. CFTR function is important to maintain cell homeostasis [138]. In ADPKD, CFTR is expressed in the apical membranes of cystic epithelial cells and is activated by cAMP, which contributes to cystic cell proliferation and chloride-dependent fluid secretion [137,139]. The role of CFTR in ccRCC remains to be determined, however, a recent study found that high *CFTR* expression in chRCC patients corresponded to a worse survival rate and suggested that *CFTR* may be involved in the progression and poor prognosis of chRCC [25]. Thus, CFTR may become a novel therapeutic target for chRCC.

In 3 to 17% of RCC patients, augmented Ca^2+^ signaling leads to hypercalcemia and shortened lifespan [140]. Increased extracellular Ca^2+^ activates several intracellular pathways through the calcium-sensing receptor (CaSR) [19]. CaSR expression in ccRCC patients positively correlated with increased MAPK and AKT signaling and higher rates of bone metastases [19].

In ADPKD, *PKD1* and *PKD2* mutations also disrupt Ca^2+^ signaling, which is thought to be a major contributor to cystic cell growth [141]. Ca^2+^ signaling is reduced in the primary cilia and endoplasmic reticulum, which elevates intracellular cAMP and fluid excretion [142]. cAMP-dependent activation of B-Raf/MEK/ERK pathway stimulates cystic cell growth [143].

## 7. Primary Cilia

Almost all mammalian cells contain non-motile primary cilia, which are evolutionary conserved organelles resembling hair-like structures or “cellular antennas”. Cilia are generated from the apical plasma membrane and employ many signaling and transport proteins [144,145,146]. Primary cilia are involved in signal transduction that regulates cell proliferation, differentiation, polarity, and tissue maintenance that include the Wnt, JAK/STAT, and mTOR pathways [144]. Inside the conduit of the renal tubule, primary cilia are thought to function in mechanosensation in response to the passing fluid, capture extracellular signals and relay them as polarity signals [146]. Therefore, ciliary dysfunction causing abnormal fluid flow in the renal tubules are expected to underlie pathological states. Because pools of cellular PC1 and PC2 interact at the cilium, primary cilia dysfunction has been linked to cyst formation in human and murine *PKD1* or *PKD2*-dependent ADPKD (reviewed in [146,147]). Tumor formation in the von Hippel-Lindau syndrome also appears linked to ciliary dysfunction [148,149]. The underlying mechanisms remain, however, unknown.

In RCC, *VHL* loss-of-function has been associated with the loss of primary cilia [149,150,151]. At the onset of ccRCC and other renal tumors, mitotic *Aurora kinase A* (*AURKA*) mRNA levels are higher than in normal tissue. AURKA is known to disrupt the assembly of the primary cilia via histone deacetylase 6 (HDAC6) activation [152]. While AURKA was originally thought to be a target of HIF-1α, where HIF-1α increases AURKA expression in the case of *VHL* deficiency, a recent study found that HIF-1α actually inhibits AURKA expression in both normal and RCC cells [149]. However, *VHL* deficiency increases β-catenin, that appears to upregulate AURKA [149]. *VHL* knock-down in hTERT RPE-1 cells resulted in fewer and shorter cilia compared to control cells [149]. Interestingly, when *VHL*-deficient cells were treated with β-catenin inhibitor iCRT14, the ciliary defects were rescued. This indicates that decreasing β-catenin activity, which in turn decreases AURKA expression, is sufficient to induce ciliogenesis when there is *VHL* deficiency [149]. As well, HIF-1α knockdown rescued the primary cilium in *VHL*-deficient cells [153].

In ADPKD cysts, AURKA is over-expressed, compared to normal renal tissues [154]. In the *Pkd1^−/−^* mouse, increased AURKA expression led to deficient ciliary resorption [155]. Loss of cilia was hypothesized to reduce cyst formation and ameliorate ADPKD, whereas cystogenesis would result from abnormal ciliary function [156,157]. However, treating the *Pkd1^−/−^* mice with alisertib, an AURKA inhibitor, lengthened the cilia compared to vehicle-treated mice, but also aggravated the rate of cystogenesis and expanded kidney volume [155]. Thus, inhibiting AURKA completely did not reduce cyst formation, but seemed to function in the opposite way. Loss of cilia has previously been shown to induce cyst formation [158]. In light of these contrasting observations, it is clear that the role of cilia in renal cystic disease and PKD needs to be further defined.

The link between cilia and PKD is tantalizing. *PKD1* and *PKD2* are two genes implicated in ADPKD, respectively encoding the PC1 and PC2 proteins that can be found in primary cilia [159,160]. When defective, the gene *Tg737^orpk^* [161] leads to ARPKD in mice; *Tg737^orpk^* encodes IFT88, a protein important for intraflagellar transport and proper assembly of the kidney cilia [162]. Immunofluorescence microscopy analyses of PC2 showed it localized in the primary cilia of human and mouse kidney cells. As well, the kidneys of *Tg737^orpk^* mutant mice had defective cilia assembly and developed cysts [159]. Compared to wild-type, cells from the *Tg737^orpk^* mice had shorter cilia yet displayed elevated PC2 immunofluorescence, meaning that IFT88 is not required for PC2 transport into the cilia [159,162]. Because of the increased PC2 signal in the mutant versus the wild-type cells, the cilia might be an important site of action for polycystins [159,162]. In addition to these constrasting results, recent observations in mice and other animal models continue to hint that the role of the cilium in renal cystic pathologies may need to be further examined. Moreover, renal tubules in zebrafish and *Drosophila* have respectively no primary cilia or no cilia, yet form renal cysts [163,164,165,166].

Interestingly, tolvaptan, an antagonist of the vasopressin V2 receptor (V2R), has been found to moderately reduce ADPKD cysts [167] and suppress ccRCC tumor growth by decreasing cell proliferation and angiogenesis, as well as increasing apoptosis [168]. In ADPKD, tolvaptan mode-of-action on ciliary signaling appears to be independent of V2R and may function through ciliary cAMP, instead of the cytoplasmic pool [169]. In this scenario, an increase in cilioplasmic cAMP, but not cytoplasmic cAMP, ultimately promotes ciliogenesis but reduces cystogenesis [169]. There are no data available on how tolvaptan may affect cilia in RCC, although we could speculate that effects would be similar.

## 8. Non-Coding RNAs

Several studies have suggested that non-coding RNA may regulate carcinogenesis, through their varied effects on cell proliferation, epithelial–mesenchymal transition, metastasis, apoptosis and/or disease progression [170,171,172]. Non-coding RNAs have roles in organismal development and their upregulated or downregulated expression may have detrimental effects on cyst formation [173] or tumorigenesis (Table 3) [172].

### 8.1. MicroRNAs

MicroRNAs (miRs) are endogenous non-coding post-transcriptional regulators with tissue-specific roles. Often used as clinical biomarkers, miRs repress translation and downregulate expression of their mRNA targets [181,184,185]. Several RCC genes are regulated by methylation, and some miRs may be involved in the epigenetic regulation of the disease (reviewed in [173]). Among the miRs known to be involved in RCC pathogenesis, the *miR-17~92* cluster, the *miR-200* family, and *miR-21* are also implicated in ADPKD.

The *miR-17~92* cluster is a set of microRNA sharing the same seed sequence to bind on the complementary nucleotides of their mRNA targets [184]. The *miR-17~92* microRNAs are upregulated in ccRCC and bind to multiple RNA targets key to ccRCC pathogenesis e.g., *VHL*, *MTOR*, *VEGF*, *HIF* [178]. Interestingly, the *miR-17~92* cluster is oncogenic in RCC [178] but appears to have tumor suppressor functions in colorectal cancers [186]. Compared to wild type kidneys, expression of the *miR-17* family is upregulated in the *Pkd1* and *Pkd2* knock out mice, respectively Ksp/Cre;*Pkd1*^F/F^ and *Pkhd1*/Cre;*Pkd2*^F/F^ [177]. The human cystic epithelium shows a similar upregulation [177]. This is due in part to the fact that the transcription factor cMyc binds to the *miR-17~92* promoter region. *miR-17~92* targets peroxisome proliferator-activated receptor (*PPAR*)-α which is normally involved in fatty acid oxidation [177,187]. It has been shown that decreased PPARα expression may accompany ADPKD pathogenesis [188].

The *Pparα* mRNA is also targeted by *miR-21* [177]. *miR-21* is used as a prognostic marker to differentiate the RCC subtypes and oncocytoma (a pre-malignant growth) because *miR-21* expression appeared highest in ccRCC compared to pRCC, chRCC, and oncocytoma tissues [179]. In *Pkd1* and *Pkd2* knock out mice, *miR-21* also targets tumor suppressor *programmed cell death 4* (*Pdcd4*) mRNA to stimulate cyst formation [180].

The *miR-200* family is highly expressed in the epithelial cells from lungs and kidney, where it is thought to prevent EMT [174,189]. In ccRCC explants, *miR-200c* inhibited EMT by repressing E-cadherin and increasing N-cadherin [176]. *miR-200* was found to bind the *Pkd1* mRNA and downregulate its expression in the Ksp/Cre;*Dicer*^F/F^ ADPKD mice [174]. This mimics the *Pkd1* loss-of-function that underpins cyst formation in ADPKD.

### 8.2. Long Non-Coding RNAs

Several long non-coding RNA (lncRNA) have been found to be dysregulated in RCC and ADPKD. The lncRNA *double homeobox A pseudogene 8* (*DUXAP8*) [181], *gastric carcinoma high expressed transcript 1* (*GHET1*) [172], *plasmacytoma variant translocation 1* (*PVT1*) [171], *HOX antisense intergenic RNA* (*HOTAIR*) [170], and *cancer susceptibility candidate 2* (*CASC2*) [182] are all linked to RCC. The lncRNA *homeobox B3 opposite strand* (*Hoxb3os*) is linked to ADPKD [183].

The lncRNA DUXAP8 was found upregulated in resected ccRCC tissue and human RCC cell lines 786-O and A498 cells (respectively derived from male and female renal cell adenocarcinoma epithelium), compared to normal kidney tissues [181]. *DUXAP8* appeared to decrease *miR-126* expression. In 786-O and ACHN cells, *miR-126* suppresses cell division [190]. In the A498 and 789-O cell lines, miR-126 inhibits expression of anti-apoptotic *cell death abnormality gene 9* (*CED*-9). The CED9 protein slows down RCC progression [181] by inducing apoptosis [191]. Therefore, increasing *DUXAP8* levels would reduce RCC progression.

Expression of the unspliced *GHET1* lncRNA positively correlated with ccRCC progression and metastasis in explanted ccRCC tissue [172]. Indicating a causal effect, *GHET1* siRNA-mediated knockdown in 786-O and A-498 cells reduced RCC cell migration and proliferation in vitro [172]. This occurred by increasing E-cadherin and promoting epithelial integrity, as well as by preventing EMT through decreased fibronectin and vimentin expression [172]. In cell culture, *GHET1* appeared to may behave as an oncogene that stabilizes and upregulates c-Myc protein expression, which enhances RCC cell proliferation [172]. To further support a role for *GHET1* in ccRCC, patients with elevated *GHET1* expression had late stage and metastatic cancer [172].

The *PVT1* lncRNA has been found overexpressed in ccRCC tissue explants, as well as in renal cancer cell lines A498, 786-O, ACHN, and Caki-1 [171]. Similar to *GHET1*, *PVT1* appeared to regulate the expression of EMT proteins, and correlated to advanced disease progression and tumor migration [171]. When *PVT1* expression was downregulated in ccRCC, caspase-3-dependent apoptosis was increased [171]. Silencing *PVT1* expression in renal cancer cell lines through *miR-16-5p* transfection restored *PVT1* normal expression levels, halted cell overproliferation, increased E-cadherin expression, and reduced expression of mesenchymal markers N-cadherin and vimentin, while promoting caspase-3 dependent apoptosis [171].

Overexpression of the *HOTAIR* lncRNA in ACHN and Caki-1 cells promoted EMT, while its inhibition by *miR-203* restored the tumorigenic effect and oncogenic potential [170]. In ccRCC tissues from patients, *HOTAIR* was overexpressed, compared to normal renal epithelial cells HK-2 [170].

The *CASC2* lncRNA appears to be downregulated in ccRCC [182]. Implicated in other cancers like endometrial and colorectal, *CASC2* normally appears to function as a tumor suppressor. *CASC2* was downregulated in ccRCC explants compared to control HEK293 cells. Restoring *CASC2* levels by transfecting cultured 786-O and A498 RCC cell lines with a vector encoding *CASC2* prevented abnormal cancer cell growth, proliferation, and migration [182].

To date, the only lncRNA with a well-defined role in ADPKD is *Hoxb3os* that is expressed specifically in the kidney [183]. A genomic screen of the lncRNA in the kidneys of ADPKD and ARPKD mice was designed to systematically target specific lncRNA and provide hypotheses on their physiological roles. The murine lines used were the Ksp/Cre (Strain 012237) control expressing Cre recombinase in the epithelial cells of the developing kidney and genitourinary tract. This line is commonly used to study the epithelial cells of the nephron. The *Pkd1*^f/f^ (Strain 010671) and *Pkd2*^f/f^ (Strain 017292) mice are ADPKD models, and the *Pkhd1*/Cre (Strain 009679) models ARPKD [183]. The lines were examined for lncRNA gene expression in different organs and 41 lncRNA were found to be common to the *Pkd1*^f/f^ and *Pkd2*^f/f^ mice. The majority of such lncRNAs seemed to affect kidney development, however, only a few appeared to promote PKD progression [183]. Expressed specifically in the mouse kidney, *Hoxb3os* was significantly downregulated in the cystic epithelium [183]. Consistently, human ortholog *HOXB-AS1* was found downregulated in the cystic kidneys of ADPKD patients [183]. *Hoxb3os* downregulation increased phosphorylation of mTORC1 and its effectors, which activated mTOR signaling, increased mitochondrial respiration and metabolism, and may contribute to renal cyst formation in ADPKD [183].

Although there have not been studies modeling lncRNA function in renal pathologies using *Drosophila*, several lncRNAs are annotated in the *Drosophila* genome [192]. Fifteen lncRNAs were found to contribute to *Drosophila* development, from embryogenesis and tissue differentiation and gene expression [193]. Other *Drosophila* lncRNAs also have roles ranging from behavioral regulation, to gonad development and sex determination [194]. Future studies will need to address the possible translational significance of these non-coding RNAs.

## 9. Drosophila Modeling for PKD

**Figure 3:**
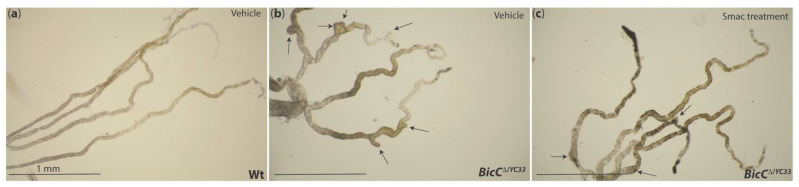
Cystic and non-cystic *Drosophila* Malpighian tubules. Malpighian tubules were micro-dissected ex vivo from synchronously cultured flies of the indicated genotype and photographed as described in [201]. (**a**) Representative Malpighian tubules micro-dissected from control wt flies treated with vehicle (water) are elongated and regularly shaped. (**b**) Tubules from *BicC* mutants (*BicC^∆/YC33^*) treated with vehicle developed several cysts (arrows), were abnormally thick and displayed apparent extra branching (lowest arrow). (**c**) *BicC^∆/YC33^* flies sibling to those in (**b**) treated with an active Smac mimetic had reduced cysts (arrows), both in number and size. *BicC^∆/YC33^* flies carry a *BicC* deletion (*Df(2L)RA5*, *∆*) in trans to a *BicC* hypomorphic allele (*BicC^YC33^*). For a discussion of the *BicC* alleles see [166]. Scale bar: 1 mm.

Particularly complex, the human kidney challenges experimentation with complex development and functional renal tubules embedded in parenchyma that hinder their molecular characterization. In contrast, the renal system of *Drosophila melanogaster* has specific and desirable features to aid the mechanistic study of renal function. *Drosophila* possesses two distinct pairs of renal (Malpighian) tubules functionally analogous to the tubular part of the human nephron (reviewed in [195]). Despite being aglomerular, the *Drosophila* renal system features separate nephrocytes that recapitulate critical function of the human glomerular podocytes (reviewed in [195]). The Malpighian tubule transcriptome is enriched in genes homologous to human renal disease genes [196]. Unlike the mammalian nephron, the fly Malpighian tubules can be exactly micro-dissected to provide pure starting material for biomolecular analyses. Combined with 75% overall genetic conservation between humans and flies [197], the genetic features and research tools that make *Drosophila* a choice model organism, such simple renal anatomy is an asset to deciphering core mechanisms of diseases originating in the renal tubule, like PKD and RCC. We have published the first-in-kind *Drosophila* model of PKD that recapitulates key physiological and molecular hallmarks of ADPKD-type cystic degeneration including *myc* and TOR activation [166]. We have found that the human *BICAUDAL C* (*BICC1*) gene functions downstream of crucial ADPKD gene *PKD1* [166]. Kidneys from *PKD1* carriers (and *Pkd1^−/−^* mice) exhibit *BICC1* loss-of-function, thus phenocopying mutational *BICC1* loss of function [166]. The cystic flies harbor mutations in the fly *BicC* ortholog [166]. *BicC* encodes an early developmental regulator of mRNA translation conserved from flies to humans ([198], reviewed in [199]). Similar to PKD-affected nephrons, the Malpighian tubules of *BicC* mutant flies displayed variably sized cysts all along, and especially in the intermediate and terminal regions (Figure 3b) [166]. The first-in-kind cystic *BicC* fly model will enable study of the molecular genetics of renal cyst formation, the precise subcellular changes occurring in the cystic cells downstream of *BicC* loss-of-function and their physiological consequences. The fly model is likely not ideal for the functional studies of the (species-specific) hormonal response to renal declining function or the ciliary role in renal cyst formation. However, that renal cysts can form in *Drosophila* corroborates the growing evidence that the role of cilia may need re-examination. Finally, the cystic flies also exhibited similar pharmacological response to rapamycin and new mimetics of the second mitochondria-derived activator of caspases (Smac) [200,201]. Discussed in more detail in Section 10.1, administration of the active Smac mimics, reduced cysts of the renal tubule both numerically and in size (Figure 3c) [200]. Note, cyst reduction can be quantified with a “cystic index” in fly and mammalian PKD models [200,201] (also see Section 10).

Interestingly, the mTOR pathway and several tumor suppressor and other genes involved in RCC and PKD are evolutionarily conserved from *Drosophila* to human (Table 4) ([202,203], reviewed in [195]). For instance, *Drosophila mTOR* (also called *dTOR*) regulates cell growth and proliferation during larval development and throughout the adult phase [204]. *dTOR* loss-of-function reduced nucleolar size, caused lipid vesicle aggregation in the larval body and cell cycle arrest [204]. *dMyc*, the *Drosophila* ortholog of proto-oncogene c-MYC, is crucial in development. dMyc regulates the cell cycle, cell proliferation, stem cell differentiation, embryo and adult size, and multiple pathways including TOR [205]. Human cMYC and fly dMyc can be functionally interchanged [206]. *dPTEN* is the *Drosophila* homolog of mammalian PTEN tumor suppressor gene, controlling cell size, cell number, and organ size during the development of multicellular organisms [207]. Suggesting that *PTEN* may function in concert with *PI3K*, the loss-of-function phenotypes of the *dPTEN* mutants were suppressed by mutations in *Dkat1* (fly AKT homolog) and *eIF4A* (fly homolog of the eponymous translation initiation factor) [207]. The *Drosophila* genome also contains homologs for key genes driving or contributing to ccRCC, including *VHL* (*dVHL*), *HIF*-*α* (*sima*), *HIF-1β* (*Tango*), and downstream effectors of the hypoxic response, *VEGF* (*Pvf1*, *Pvf2*, *Pvf3*), *EGFR* (*dEGFR*), and *GLUT1* (*dGlut1*) (Table 4). Several of these genes are known to have developmental functions (Table 4) and some are reportedly expressed in the Malpighian tubules (e.g., *dVHL*, *Pvf1*, *Pvf2*, *Pvf3*, and *dEGFR* [192]) but their contribution to renal physiology needs to be characterized. Functional studies in *Drosophila*, however, could provide important insight into their potential contribution to renal physiology and help decipher their core molecular function. Similar to its human homolog, *Drosophila VHL* functions in the systemic hypoxic response [208]. It also presides to tracheal and vascular development largely independently of *HIF*/*sima* ([209,210], reviewed in [211]) which suggests that *dVHL* has several functions. *dVHL* mutation upregulates FGFR signaling through Breathless, which is needed for proper tracheal branching (reviewed in [212]). In the tracheal epithelium of *dVHL* mutants, the Breathless/FGFR protein accumulates at the cell surface due to defective endocytosis [210]. This led to the discovery that *dVHL* genetically interacts with the dynamin homolog *shibire* and the fly homolog of human metastasis suppressor *NME1/2* called *abnormal wing discs* (*awd*) [213]. Indicating roles in fundamental epithelial biology, *dVHL* is also important to regulate microtubule stability during morphogenesis of the follicular epithelium enveloping the egg chambers [214]. Importantly, discoveries in the fly model provided functional clues on mammalian VHL. Both a Cre/lox *Vhl* null mouse model and organoids confirmed the *Vhl* morphogenetic role in renal tubules and its involvement of the endocytic pathway in regulating FGFR signaling [210]. To probe potential for pinpointing “first hit” changes predisposing to cancer, one study reported the differential gene expression in Malpighian tubules from wild type and *VHL^+/−^* heterozygotes, that were regarded as mimics of the frequent first-hit events in ccRCC [215]. Reportedly, patterns of relative RNA perturbation in the fly indicated changes in phosphatases, that may indicate altered signal transduction and were similar to primary cell cultures from RCC patients [216]. *VHL* haploinsufficiency, or the reduction of *VHL* dose, as seen upon “first hit”, is not sufficient to fuel cancer, however, it shifts metabolism by activating some of the Warburg effect factors and some of the mediators of glutamine reductive metabolism, effectively placing the cells on the path to transformation [216]. Moreover, upregulation of oxidative stress factors and histone acetylation enzymes combined with the downregulation of genes involved in the DNA damage response, cell cycle arrest, apoptosis and growth factor response also endow cells with properties conducive to transformation [216]. However, crucial ccRCC characteristics, e.g., constitutively active NF-κB, are not achieved upon *VHL* “first-hit” [216,217]. Consistently, *VHL* haploinsufficiency is associated with precancerous lesions e.g., colon polyps and skin cancers [216]. With this knowledge, the gene expression changes that accompany ccRCC transformation could be effectively modeled in *Drosophila*, where mechanistic detail can be dissected using powerful collections of genetic mutants and tissue-specific drivers allowing knock-down and over-expression of specific genes, regardless of their genomic location. *dVHL* heterozygotes could be used to mimic subsequent mutational hits in the second *VHL* copy employing tissue-specific knockdown to better model somatic mutations. Note, *Drosophila dVHL^1.1^* homozygotes survive until mid-larval stages [210]. Similarly, RNAi-induced knockdown could be used to target other genes that are found co-mutated in ccRCC to evaluate their contribution to eventual cell transformation and their potential prognostic value. Indeed, homologs of the RCC genes encoding chromatin modifiers and epigenetic factors are also conserved to *Drosophila* (Table 4). “Humanized flies” expressing the human homologs could be generated by transgenesis, their functionality assessed and their mechanistic role deciphered. Effectiveness of targeting single or simultaneously multiple pathways that are dysregulated in ccRCC could be time- and cost-effectively determined in flies, providing translational information for therapeutic design. Patient-specific variants could be expressed in flies to generate “avatars” for tailored drug screening in personalized medicine approaches [218]. The emerging potential for fly models to guide pharmacological applications is discussed in Section 10. Aspects of ccRCC linked to species- and individual-specific immunological response would be much more difficult to model in the fly. However, such studies may benefit from improved mechanistic knowledge of the pathways underpinning RCC. Altogether, the conservation of factors involved in human renal neoplasias in *Drosophila* suggests feasibility of modeling critical aspects of RCC in the fly to obtain mechanistic insights into disease pathology and determine treatment potential.

## 10. Pharmacological Strategies

The HIF/VEGF and mTOR pathways have been exploited for therapeutic purpose in ccRCC and their modulation appeared promising (reviewed in [242]). mTOR inhibitors everolimus and temsirolimus have been approved for treatment of advanced RCC due to their antitumor activity in clinical trials, although temsirolimus exhibited limited efficacy [243,244,245,246]. Several other drug treatments for RCC are listed in Table 5. Of note, several of these treatments target pathways conserved from humans to *Drosophila*. Similar to ccRCC, mTOR inhibition with rapamycin, and derivatives everolimus and sirolimus, was found to reduce ADPKD cysts in patient and several models, including *Drosophila* [102,104,166,247,248,249,250]. Despite its promising results in animal models, mTOR inhibition by rapamycin and its derivatives, called rapalogs, was much less effective in patients, may elicit severe side effects ([251], reviewed in [252]) and offer only temporary improvements which is unsuitable to the therapy of a chronic disease such as ADPKD. Without precise molecular characterization of the pathological mechanisms of both ccRCC and ADPKD, it is imperative to search for predictive biomarkers, targets, and effective therapeutics [11]. Using a comparative approach in the study of ccRCC and ADPKD may allow to leverage upon their similarities to improve our mechanistic knowledge and determine potential remedial interventions and drug repurposing.

RCC characteristically displays drug resistance and is regarded as an immunogenic tumor [263], thus it is a good candidate for immunotherapy. Although not easily transferrable to the fly model, we discuss this important approach for completeness. Immune dysfunction has been found to promote RCC tumor growth and invasion (reviewed in [263]). A novel immune regulating agent, programmed death receptor (PD)-1 is a promising candidate promoting antitumor immunity [263,264]. PD-1 is an immunoinhibitory receptor that is not normally found in healthy kidneys but is significantly expressed in primary and metastatic ccRCC [265,266,267]. PD-1, and its ligand PD-L1, exert their inhibitory activity during T-cell activation in tumors which deflects T-cell mediated tumor targeting [263]. The PD-1 pathway blockade with several agents such as nivolumab and pembrolizumab bypasses T-cell inhibition, leading to increased T-cell proliferation, and resulting in continued antitumor response in patients with advanced ccRCC (reviewed in [263]). Therefore, PD-1 pathway activation appears a prognostic marker as well as a promising immunotherapeutic method, despite its well described limitations due to tumor heterogeneity, different positivity thresholds, as well as the complex relationship between tumors and the immune system [268,269].

### 10.1. Smac Treatment

**Figure 4:**
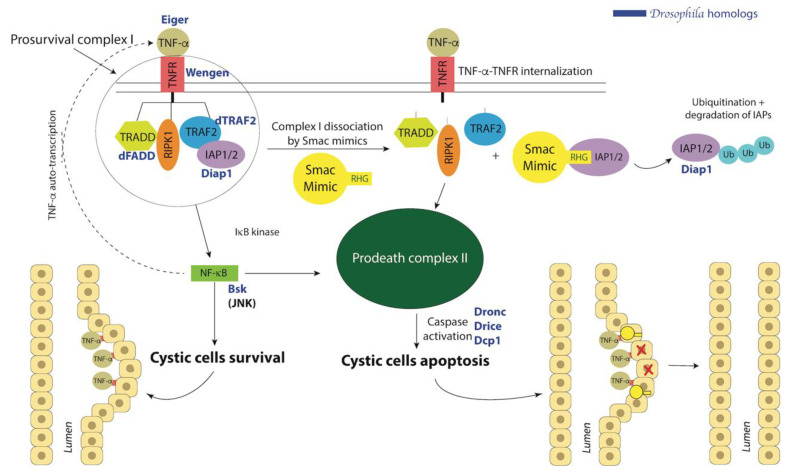
TNF-α-dependent Smac mimicry in ADPKD with corresponding *Drosophila* homologs. ADPKD cystic cells exhibit upregulated TNF-α and TNF receptor TNFR1. TNF-α-TNFR signaling leads to TNF-α auto-transcription and pro-survival signals through complex I (left), which promotes cyst growth. Smac mimetics (or mimics) target and sequester inhibitor of apoptosis proteins (IAPs), which leads to the dissociation of complex I and the formation of the pro-death complex II to induce apoptosis of cystic cells (right). *Drosophila homologs* are added to show the potential for modeling. See text for more details. Modified from [279].

Apoptosis is often downregulated in cancers, fueling the abnormal tumorigenic growth. Thus, restoring the defective apoptosis is one therapeutic strategy in oncology. One approach that holds promise in both ccRCC and ADPKD, is that mediated by a class of molecules derived from Smac, (also known as direct inhibitor of apoptosis-binding protein with low pI, DIABLO), called Smac mimetics [270]. Following a pro-apoptotic stimulus (e.g., tumor necrosis factor (TNF)-α signaling) several pro-apoptotic factors, including Smac, are released from the mitochondria into the cytosol, thereby initiating proapoptotic cascades [271]. Smac binds to and sequesters inhibitor of apoptosis proteins (IAPs) and displaces them from initiator caspase-9 (Figure 4). This activates caspase-9 and promotes cleavage of downstream effector caspases driving apoptosis [272,273]. Smac mimicry has previously been investigated in oncology for induction of apoptosis in TNF-α-dependent cancers (reviewed in [274,275]). In the first study investigating Smac expression in RCC, primary cells from patients and cell lines NC65, ACHN, and Caki-1, quantitative immunoblot analysis revealed four-fold lower Smac levels in RCC compared to normal kidneys [276]. Moreover, expression of Smac inversely correlated with disease progression and RCC tumor grade, while patient survival positively correlated with residual Smac expression levels [276]. Consistently, it was found that patients with metastatic ccRCC had lower Smac expression than patients with primary localized RCC [276,277,278]. These results suggest that Smac expression levels could be used as a prognostic tool for RCC and potentially as therapeutic target. Smac mimetic LCL161 is being tested in RCC as part of broader clinical trial (NCT02890069) for colorectal cancer, non-small cell lung cancer, and negative breast cancer.

Smac/DIABLO has also been shown to be a promising treatment for ADPKD. In a Pkd1^−/−^ mouse model, Smac-mimetic GT13072 was found to induce cystic cell death in a TNF-α-dependent manner [280]. The rationale of Smac mimicry in ADPKD therapy relies on the fact that the cystic fluid of ADPKD cysts (in both the Pkd1^−/−^ mouse and ADPKD patients) contains high amounts of TNF-α and the cells lining the cyst also overexpress the TNF-α receptor 1, which activates TNF-α signaling [280,281]. Conversely, non-cystic cells do not exhibit TNF-α pathway activation. Thus, the tubular cells lining the ADPKD cyst are sensitized to Smac mimicry, unlike the neighboring non-cystic cells [280]. As expected, the Smac-mimetic GT13072 was found to reduce cysts in the Pkd1^−/−^ mouse model [280]. The TNF pathway is highly conserved in Drosophila (Figure 4) [282,283,284,285]. Therefore, we hypothesized that Smac may induce cystic cell death via the activation of TNF-α-dependent apoptosis, similar to the Pkd1^−/−^ mouse model [280]. As a proof of principle, we tested four new Smac mimetics for their cyst-reducing potential in the Drosophila PKD model [166,200]. Note, the fly PKD model displayed conserved response to rapamycin, hinting at conservation of core molecular mechanisms of cyst formation [166]. For the cystic analysis, newly hatched BicC mutant flies were fed either the Smac mimetics (20 μM) for 20 days or vehicle (water). For each treatment, Malpighian tubules from 50 female flies were micro-dissected ex vivo (a representative example is shown in Figure 3) and the number of cysts was scored, mapping the cysts to the different regions of the Malpighian tubules, and distinguishing between anterior and posterior tubules [166,200]. Results were then analyzed statistically to derive a “cystic index” used to compare treatments [200]. For a discussion of the cystic index in the fly model, see [201]. Upon Smac treatment, the BicC mutants displayed a significant overall reduction of cysts, and differential compound efficacy [200]. Interestingly, the Smac mimics displayed selectivity for distinct regions of the renal tubule, that if conserved to humans, may offer opportunity for precise targeting in personalized medicine [200]. While the mechanistic detail of Smac mimicry in the Drosophila model and in human ADPKD remains to be determined, the conservation of the pharmacological response between flies and humans suggests that Drosophila genetics and drug assays may be combined to accelerate progress in this direction.

An interesting similarity between ccRCC and ADPKD, it was found that TNF-α levels significantly increase as the stages of RCC progress [286,287]. TNF-α was overexpressed in high-grade ccRCC tissues and tumor-associated macrophages [288]. Moreover, TNF-α levels positively correlated with ccRCC cell invasion and the epithelial-mesenchymal transition of ccRCC cells in vitro [289]. Overall, TNF-α appears a key player in tumor invasion and metastasis in ccRCC [288]. Besides its expected value as a possible prognostic marker for ccRCC, these results strongly suggest potential for Smac (potentially combination) treatment to limit tumor cell invasion and metastasis in ccRCC.

### 10.2. Melatonin Treatment

Melatonin is a ubiquitous neurohormone secreted at night from the pineal gland and is mainly thought to function within the circadian rhythm (reviewed in [290,291]). In murine models and Drosophila, melatonin has been found to increase lifespan, while treating age-related diseases [292,293,294,295,296,297]. It has also been widely employed in oncology for as its oncostatic effects in many cancer types ([298], reviewed in [299,300]). Melatonin has been shown to function at multiple levels to halt cancer cell proliferation via the mTOR, MAPK, EGF (and other) pathways responding to several growth factors (reviewed in [300]).

In a few studies, melatonin was found to promote maintainance of proper kidney function [301,302,303]. In ccRCC, melatonin has been shown to reduce metastasis by suppressing the Akt-MAPK cascade, NF-kB DNA-binding activity, and matrix metalloproteinase (MMP)-9 transactivation [304]. As well, melatonin induced cell death in the renal cancer cell line Caki, which occurred via upregulation of the E2F1 and Sp1 transcription factors, and increased expression of the Bcl-2-interacting mediator of cell death (Bim) through transcriptional and post-transcriptional mechanisms [305,306]. Melatonin may hold promise in ADPKD therapy as well. Very recently, we published that melatonin treatment significantly reduced renal tubule cysts in the Drosophila PKD model [307]. In this study, nightly administration of 150 μM melatonin effectively decreased the cystic index of BicC flies, further suggesting the conservation of common core mechanisms of abnormal cystic and neoplastic cell growth [307]. While the cyst-reducing mechanisms of melatonin are being investigated, melatonin holds special interest as a molecule to treat PKD because its lack of toxicity, high tolerability, and potential for combination therapy would be perfectly suited to the protracted treatment of chronic PKD.

## 11. Discussion

Abnormal neoplastic growth in ccRCC and ADPKD appears to result from the accumulation of several mutational hits that cooperate to change fundamental cell properties, metabolism, and behavior. Among cancers, ccRCC has a characteristic mechanism of oncogenesis resulting from high genomic instability and the unique accumulation of several mutations that affect the cancer outcomes [15,21]. Consistent with such genetic instability, ccRCC is largely sporadic. Benign ADPKD has a component of dominant genetic inheritance through the *PKD1* and *PKD2* genes, but a second-hit model may best explain the breadth of clinical observations ([308,309,310], reviewed in [34]). In part because they affect the same tubular cells, ccRCC and ADPKD share similar alterations in proteins and non-coding RNAs with proto-oncogene and tumor suppressor properties that affect the replicative capacity of the mutated cells and substantially change their metabolic profile. Co-contributing mutations complicate experimentation. However, similarities between ccRCC and ADPKD have raised significant interest because they might be leveraged to advance knowledge of neoplastic growth and to determine key differences between cancerous growth in ccRCC and benign ADPKD [311,312,313]. Simpler models like *Drosophila* have been particularly successful to decipher core disease mechanisms. Relevant to oncology, the first observations of cancer in *Drosophila* date back to Mary Stark in 1918 [314,315,316]. The concept of cooperative oncogenesis also emerged in *Drosophila* studies [317]. Epithelial tumor formation reviewed in [318], cell motility and metastasis [319,320], context-dependent tumorigenesis [321], stress signaling in cooperative oncogenesis [322], cell polarity [323,324,325], cell competition [326], and the Warburg effect [327] are but a few examples of successful application of fly genetics to decipher key mechanisms of neoplastic growth and cancer.

At the cellular level, ccRCC and ADPKD display several dysfunctional signal transduction pathways, metabolic remodeling and degrees of genetic instability. This makes them particularly challenging to study and complicates therapeutics. Studying the homologous pathways using the extensive collection of *Drosophila* genetic tools may provide valuable insight into both diseases. Genetic knockdown in *dVHL* heterozygotes may help understand the progression from “first hit” predisposition to cell transformation, and the effects of concurrent mutations resulting from ccRCC genetic instability at the cellular and physiological levels, as well as identify crucial pathways to target therapeutically.

The similarities between RCC and ADPKD appear to be also reflected pharmacologically at least in the case of rapalogs, Smac mimetics and melatonin. Rapalogs have proven particularly effective and have been approved for use in RCC therapy for the few cases featuring MTOR activation [243,244,245,246]. Rapamycin and derivatives have also shown promising results in ADPKD and ARPKD models, yet they are not suitable PKD therapeutics because of progressive loss of efficacy and long-term toxicity [102,104,166,247,248,249,250,251,252]. Smac mimetics and melatonin also appear to be effective in RCC and ADPKD models and are expected to present at least partially distinct mechanisms of action. Such underlying similarities may be leveraged to identify novel drug candidates that could be repurposed for RCC and/or ADPKD therapy. Such strategy is expected to be particularly beneficial to ADPKD, for which therapeutic options are severely limited [311]. Moreover, knowledge “spill-over” of potentially effective pharmacological options may help identify candidates for combination therapy. While the effects of the tumor microenvironment and immunity may be challenging to model in flies, *Drosophila*, has established value toward developing mechanistic knowledge of core diseased pathways. Such knowledge in turn will facilitate investigation of the tumor microenvironment and immunity in mammalian systems. Still a relatively untapped resource in drug studies, robust pathway conservation between *Drosophila* and humans and effective transgenesis enabling expression of human proteins and variants in the fly, empower pharmacological studies with translational significance. This has been particularly successful in oncology ([328,329], reviewed in [330]) and may have potential for ADPKD [200,201,307]. Disease modeling relies on models for discovery science, preclinical models of diseased states, and models for therapeutic outcomes. Leveraging on the contemporary comparative knowledge and employing experimental setups aware of the phylogenetic differences, *Drosophila* may become especially beneficial to accelerate basic and clinical progress for diseases with complex and challenging cooperative mechanisms and scarce therapeutical options such as ccRCC and ADPKD.

## Figures and Tables

**Table 1:** Renal cell carcinoma (RCC) subtypes and presentation.

RCC Subtypes	Prevalence	Description	Gene Mutations	References
Clear cell RCC	70–90% of all RCC 1–3% of all malignant visceral neoplasms	Tumor cells with clear cytoplasm	*VHL, PBRM1, SETD2, BAP1, MTOR, TCEB1, PIK3CA, KDM5C. TP53, PTEN*	[5]
Papillary RCC Type 1	10–15%	Papillae lined with one layer of tumor cells with low grade nuclei and partially clear cytoplasm	*MET*	[5,20,21]
Papillary RCC Type 2	10–15%	Abundant eosinophilic cytoplasm and large pseudostratified cells with high grade nuclei	*CDKN2A, SETD2, NRF2, FH*	[20,21,22]
Chromophobe	5–7%	Large cells, polygonal reticulated cytoplasm, distinct cell borders, atypical nuclei with perinuclear halo. Contain fine eosinophilic granules.	*TP53, PTEN, TSC1*	[21,23,24,25]
Multilocular Cystic RCC (Cystic-solid)	1–5%	Multiseptated cystic space, cysts lined by cuboidal clear cells or flattened epithelium with septa and clear cytoplasm	*GIGYF2, FGFR3, SETD2, BCR, KMT2C, TSC2*	[26,27]
Bellini Duct Carcinoma	Less than 1% of all malignant kidney tumors	Malignant tumor containing metanephric, stromal and epithelial derivatives	*CDKN2A* deletion, *SLC* gene altered	[28]
Von Hippel Lindau disease	1% of RCC	Visceral cysts in kidney, pancreas, epididymis	*VHL, MYC* as potential target of 8q amplification.	[29]
Wilm’s tumors (nephroblastoma)	5–6% of kidney cancer in children	Triphasic. Composed of epithelial, blastemal, and stromal elements	*WT1, CTNNB1, AMER1* Predisposition genes identified including *TRIM28, FBXWJ, NYNRIN, KDM3B*	[30,31]

**Table 2:** Fuhrman nuclear grade classification for RCC tumors.

Tumor Grade	Morphology	Prevalence	Description
G1	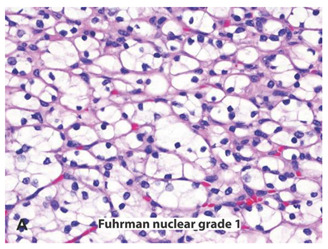	Very rare	Round or uniform nuclei with barely visible nucleoli
G2	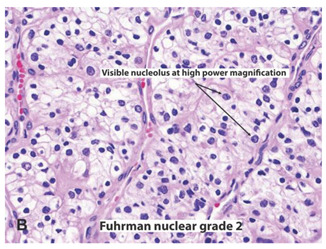	40%	Somewhat irregular nuclear contours with nucleoli visible only at 400×
G3	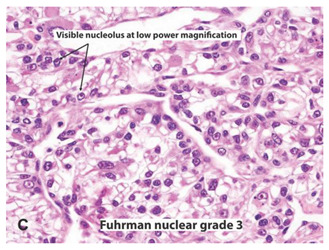	30–40%	Moderate to prominent irregular nuclear contours with nucleoli visible at 100×
G4	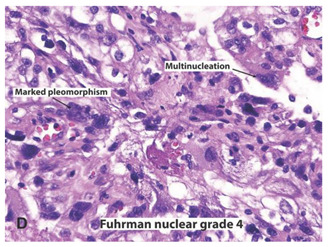	15%	Multilobular and grotesque nuclei with large and prominent nucleoli

Images reprinted with permission from Gladell P. Paner (American Urology Association). Text has been redrawn. References: [32,33].

**Table 3:** Role of Non-Coding RNA Involved in RCC and PKD.

Non-Coding RNA	Expression	Role	Model	References
*miR-200* family	Downregulated in cystic kidneys	Promote cyst formation by binding to *Pkd1*	Ksp/Cre; *Dicer*^F/F^ mutant mice	[174]
	Downregulated in ccRCC	Inhibits EMT	ccRCC tissues from patients	[175,176]
*miR-17~92* cluster	Upregulated in ADPKD	Targets *Pparα* to promote cyst formation	Ksp/Cre;*Pkd1*^F/F^ (*PKD1*-KO) and Pkhd1/Cre;P*kd2*^F/F^ (*Pkd2*-KO) mice kidneys	[177]
	Upregulated in ccRCC	Promote tumor cell proliferation	Kidney tissues from ccRCC patients	[178]
*miR-21*	Upregulated in RCC	Prognostic marker	RCC tissues from patients	[179]
	Upregulated in PKD	Promote cyst formation	Pkhd1/Cre;*Pkd2*^F/F^, Ksp/Cre;*Pkd1*^F/F^, Ksp/Cre;*Hnf-1β*^F/F^	[180]
*DUXAP8*	Upregulated in RCC	Decreases *miR-126*	Renal cancer cell lines A498, 786-O	[181]
*GHET1*	Upregulated in RCC	Promotes EMT	RCC tissues form patients, 786-O and A-498 cells	[172]
*PVT1*	Upregulated in RCC	Decreases *miR-16-5p* expression	ccRCC tissues from patients, renal cancer cell lines A498, 786-O, ACHN, Caki-1	[171]
*HOTAIR*	Upregulated in RCC	Attenuated by *miR-203*	ccRCC from patients, renal cancer cell lines ACHN, Caki-1	[170]
*CASC2*	Downregulated in RCC	Targeted by *miR-21*	RCC tissues from patients, renal cancer cell lines A498, 786-O	[182]
*Hosb3os*	Downregulated in ADPKD	Negatively regulates mTOR signaling	Ksp/Cre;*Pkd1*^f/f^ and Pkhd1/Cre;*Pkd2*^f/f^ mice, mIMCD3 cells	[183]

**Table 4:** *Drosophila melanogaster* RCC and PKD gene homologs and related functions.

Human RCC Gene	*Drosophila* Homolog	Mutant Phenotype(s)	Molecular and Cellular Function(s)	Reference(s)
*VHL*	*dVHL*	Cell migration and polarity defective	Regulates embryonic morphogenesis of trachea and follicular epithelium; promotes endocytic vesicle transport	[210,219]
*HIF-* *α*	*sima*	Exacerbated hyperlactatemic phenotype	During development, directs hypoxia-driven terminal branching of trachea	[220,221]
*HIF-1* *β*	*Tango*	CNS midline and tracheal defects	Role in CNS midline development and tracheal tubule formation; possibly a cytosolic sink to sequester Sima and prevent interaction with Notch	[222,223]
*VEGF*	*Pvf1* *Pvf2* *Pvf3*	Severe defects in hemocyte migration	Regulation of blood cell migration and activation of the canonical Ras/Raf/MAPK cascase, PI3K, TORC1, Rho family of small GTPases, and JNK cascade	[224,225,226]
*EGFR*	*dEGFR*	Glial hyperplasia, CNS morphogenesis defects, lethal	Growth regulation, cell survival and proliferation, developmental patterning	[227,228]
*GLUT1*	*dGlut1*	-	Glucose transport/uptake in neurons	[229]
*PBRM1*	*polybromo*	-	Chromatin remodeling with Brahma complex; regulation of gene transcription	[230]
*BAP1*	*Calypso*	-	With ASX, forms a Polycomb group (PcG) protein complex called PR-DUB involved in deubiquitination; required for efficient activity on nucleosomes	[231,232]
*SETD2*	*Set2*	-	Encodes an essential histone methyltransferase that functions with CG4747 to facilitate targeting of the male-specific lethal (MSL) complex to active genes	[233]
*mTOR*	*dTor*	Reduced nucleolar size, lipid vesicle aggregation in larval fat body, cell type-specific pattern of cell cycle arrest	Regulates cellular growth; amino acid and nutritional sensing	[204,234]
*c-Myc*	*dMyc*	Smaller cell size, body size, and viability	Regulates cell cycle, stem cell differentiation, cell proliferation, embryo and adult size	[205,235]
*PTEN*	*dPTEN*	Larger eyes and heads, faster proliferation	Encodes a negative effector of insulin signaling; control of cell size, proliferation and apoptosis	[207,236,237]
*TP53*	*Dp53*	Massive cell death in developing eye; apoptotic defect of primordial germ cells	Adaptive responses to genotoxic stress; inhibits cell differentiation; activates canonical caspase-dependent apoptosis pathway during stress	[238,239,240]
*TCEB1*	*EloB*	Vein truncation in wings	Essential gene that facilitates Elongin complex assembly and stability; important for wing development and identity	[241]

**Table 5:** Drug treatments for RCC.

Drugs	Targets	References
Sunitinib	Inhibits several receptor tyrosine kinases, including VEGFR types 1 and 2, PDGFRs, and more.	[253]
Axitinib	Potent inhibitor of VEGFR1,2 and 3. Axitinib had a higher progression free survival (defined as the range between the date of the first dose and the date of disease progression or death) compared to sorafenib. Axitinib established the utility of second-generation angiogenesis inhibitors with broader activity to overcome sunitinib resistance.	[254]
Sorafenib	Multi-targeted tyrosine kinase inhibitor of VEGFR2, VEGFR3, and PDGFR-β.	[255,256]
Lenvatinib	Selectively targets VEGFR1, 2, and 3, FGFRs, and PDGFR-α. Also effective towards non-VEGFR pathways.	[257]
Lenvatinib + everolimus	Combination exhibited longer progression free survival than everolimus (but not lenvatinib) alone.	[258,259]
Trebananib	Fusion protein disrupting the interaction of angiopoietin 1 and 2 with receptor Tie2. Did not show promising effects in anti-angiogenesis resistant RCC.	[260]
Dalantercept	Antagonist of Activin receptor-like kinase (ALK)-1/Bone morphogenetic protein (BMP)-9 signaling for the treatment of metastatic RCC. Combination with axitinib in heavily pretreated ccRCC patients did not seem to improve.	[261,262]

## References

[1] Yao Y., Dai W. (2014). Genomic instability and cancer. J. Carcinog. Mutagen..

[2] Chow W.H., Dong L.M., Devesa S.S. (2010). Epidemiology and risk factors for kidney cancer. Nat. Rev. Urol..

[3] Znaor A., Lortet-Tieulent J., Laversanne M., Jemal A., Bray F. (2015). International variations and trends in renal cell carcinoma incidence and mortality. Eur. Urol..

[4] Ljunberg B., Campbell S.C., Choi H.Y., Jacqmin D., Lee J.E., Weikert S., Kiemeney L.A. (2011). The epidemiology of renal cell carcinoma. Eur. Urol..

[5] Hsieh J.J., Purdue M.P., Signoretti S., Swanton C., Albiges L., Schmidinger M., Heng D.Y., Larkin J., Ficarra V. (2017). Renal cell carcinoma. Nat. Rev. Dis. Primers.

[6] Amiji M.M., Ramesh R. (2018). Diagnostic and Therapeutic Applications of Exosomes in Cancer.

[7] Siegel R.L., Miller K.D., Fuchs H.E., Jemal A. (2021). Cancer Statistics, 2021. Cancer J. Clin..

[8] Capitanio U., Bensalah K., Bex A., Boorjian S.A., Bray F., Coleman J., Gore J.L., Sun M., Wood C., Russo P. (2019). Epidemiology of renal cell carcinoma. Eur. Urol..

[9] Moch H., Cubilla A.L., Humphrey P.A., Reuter V.E., Ulbright T.M. (2016). The 2016 WHO classification of tumours of the urinary system and male genital organs—Part A: Renal, penile, and testicular tumours. Eur. Urol..

[10] Haake S.M., Rathmell W.K. (2017). Renal cancer subtypes: Should we be lumping or splitting for therapeutic decision making?. Cancer.

[11] Wolf M.M., Rathmell W.K., Beckermann K.E. (2020). Modeling clear cell renal cell carcinoma and therapeutic implications. Oncogene.

[12] Muglia V.F., Prando A. (2015). Renal cell carcinoma: Histological classification and correlation with imaging findings. Radiol. Bras..

[13] Kovacs G., Akhtar M., Beckwith B.J., Bugert P., Cooper C.S., Delahunt B., Eble J.N., Fleming S., Ljungberg B., Medeiros L.J. (1997). The Heidelberg classification of renal cell tumours. J. Pathol..

[14] Linehan W.M., Walther M.M., Zbar B. (2003). The genetic basis of cancer of the kidney. J. Urol..

[15] Manley B.J., Hakimi A.A. (2016). Molecular profiling of renal cell carcinoma: Building a bridge towards clinical impact. Curr. Opin. Urol..

[16] Zekri J., Ahmed N., Coleman R.E., Hancock B.W. (2001). The skeletal metastatic complications of renal cell carcinoma. Int. J. Oncol..

[17] Janzen N.K., Perry K.T., Schulam P.G. (2003). Laparoscopic radical nephrectomy and minimally invasive surgery for kidney cancer. Kidney Cancer.

[18] Lam J.S., Klatte T., Kim H.L., Patard J.J., Breda A., Zisman A., Pantuck A.J., Figlin R.A. (2008). Prognostic factors and selection for clinical studies of patients with kidney cancer. Crit. Rev. Oncol. Hematol..

[19] Frees S., Breuksch I., Haber T., Bauer H.K., Chavez-Munoz C., Raven P., Moskalev I., Costa N.D., Tan Z., Daugaard M. (2018). Calcium-sensing receptor (CaSR) promotes development of bone metastasis in renal cell carcinoma. Oncotarget.

[20] Warrick J.I., Tsodikov A., Kunju L.P., Chinnaiyan A.M., Palapattu G.S., Morgan T.M., Alva A., Tomlins S., Wu A., Montgomery J.S. (2014). Papillary renal cell carcinoma revisited: A comprehensive histomorphologic study with outcome correlations. Hum. Pathol..

[21] Durinck S., Stawiski E.W., Pavia-Jimenez A., Modrusan Z., Kapur P., Jaiswal B.S., Zhang N., Toffessi-Tcheuyap V., Nguyen T.T., Pahuja K.B. (2015). Spectrum of diverse genomic alterations define non-clear cell renal carcinoma subtypes. Nat. Genet..

[22] Delahunt B., Eble J.N. (1997). Papillary renal cell carcinoma: A clinicopathologic and immunohistochemical study of 105 tumors. Mod. Pathol..

[23] Shuch B., Amin A., Armstrong A.J., Eble J.N., Ficarra V., Lopez-Beltran A., Martignoni G., Rini B.I., Kutikov A. (2015). Understanding pathologic variants of renal cell carcinoma: Distilling therapeutic opportunities from biologic complexity. Eur. Urol..

[24] Sakamoto H., Yamasaki T., Sumiyoshi T., Utsunomiya N., Takeda M., Kamba T., Nakamura E., Ogawa O. (2018). A family case with germline TSC1 and mtDNA mutations developing bilateral eosinophilic chromophobe renal cell carcinomas without other typical phenotype of tuberous sclerosis. J. Clin. Pathol..

[25] Wang S., Yu Z.H., Chai K.Q. (2019). Identification of CFTR as a novel key gene in chromophobe renal cell carcinoma through bioinformatics analysis. Oncol. Lett..

[26] Chowdhury A., Chakraborty D., Bhattacharya P., Dey R. (2013). Multilocular cystic renal cell carcinoma a diagnostic dilemma: A case report in a 30-year-old woman. Urol. Ann..

[27] Kim S.H., Park W.S., Chung J. (2019). SETD2, GIGYF2, FGFR3, BCR, KMT2C, and TSC2 as candidate genes for differentiating multilocular cystic renal neoplasm of low malignant potential from clear cell renal cell carcinoma with cystic change. Investig. Clin. Urol..

[28] Wang J., Papanicolau-Sengos A., Chintala S., Wei L., Liu B., Hu Q., Miles K.M., Conroy J.M., Glenn S.T., Costantini M. (2016). Collecting duct carcinoma of the kidney is associated with CDKN2A deletion and SLC family gene up-regulation. Oncotarget.

[29] Maher E.R., Neumann H.P., Richard S. (2011). Von Hippel-Lindau disease: A clinical and scientific review. Eur. J. Hum. Genet..

[30] Breslow N.E., Beckwith J.B. (1982). Epidemiological features of Wilms’ tumor: Results of the National Wilms’ Tumor Study. J. Natl. Cancer Inst..

[31] Weksberg R., Brzezinski J. (2019). Identifying new Wilms tumour predisposition genes. Lancet Child Adolesc. Health.

[32] Gladell P., Paner M.D. Clear Cell Renal Carcinoma: Fuhrman Nuclear Grade. https://www.auanet.org/education/auauniversity/education-products-and-resources/pathology-for-urologists/kidney/renal-cell-carcinomas/clear-cell-renal-cell-carcinoma-fuhrman-nuclear-grade.

[33] Williamson S.R. Grading. https://www.pathologyoutlines.com/topic/kidneytumormalignantnucleargrading.html.

[34] Bergmann C., Guay-Woodford L.M., Harris P.C., Horie S., Peters D.J.M., Torres V.E. (2018). Polycystic kidney disease. Nat. Rev. Dis. Primers.

[35] Sparapani S., Millet-Boureima C., Oliver J., Mu K., Hadavi P., Kalostian T., Ali N., Avelar C.M., Bardies M., Barrow B. (2021). The biology of vasopressin. Biomedicines.

[36] Harris P.C., Torres V.E. (2009). Polycystic kidney disease. Annu. Rev. Med..

[37] Ong A.C.M., Harris P.C. (2005). Molecular pathogenesis of ADPKD: The polycystin complex gets complex. Kidney Int..

[38] Neumann H.P.H., Zbar B. (1997). Renal cysts, renal cancer and von Hippel-Lindau disease. Kidney Int..

[39] Bonsib S.M. (2009). Renal cystic diseases and renal neoplasms: A mini-review. Clin. J. Am. Soc. Nephrol..

[40] Chen S., Jin B., Xu L., Fu G., Meng H., Liu B., Li J., Xia D. (2014). Cystic renal cell carcinoma: A report of 67 cases including 4 cases with concurrent renal cell carcinoma. BMC Urol..

[41] Zhang J., Liu B., Song N., Hua L., Wang Z., Gu M., Yin C. (2013). Diagnosis and treatment of cystic renal cell carcinoma. World J. Surg. Oncol..

[42] Ishikawa I., Saito Y., Onouchi A., Kitada H., Suzuki S., Kurihara S., Yuri T., Shinoda A. (1980). Development of acquired cystic disease and adenocarcinoma of the kidney in glomerulonephritic chronic hemodialysis patients. Clin. Nephrol..

[43] Grantham J. (1990). Polycystic kidney disease: Neoplasia in disguise. Am. J. Kidney Dis..

[44] de Werra C., Donzelli I., Perone M., Micco R.D., Orabona G. (2009). Multifocal and multicentric tumors. Multiple Primary Malignancies. Updates in Surgery.

[45] Nargund A.M., Pham C.G., Dong Y., Wang P.I., Osmangeyoglu H.U., Xie Y., Aras O., Han S., Oyama T., Takeda S. (2017). The SWI/SNF Protein PBRM1 Restrains VHL-Loss-Driven Clear Cell Renal Cell Carcinoma. Cell Rep..

[46] Drusian L., Boletta A. (2019). MTORC1-driven accumulation of the oncometabolite fumarate as a potential critical step in renal cancer progression. Mol. Cell. Oncol..

[47] Shao X., Somlo S., Igarashi P. (2002). Epithelial-specific Cre/lox recombination in the developing kidney and genitourinary tract. J. Am. Soc. Nephrol..

[48] Mandriota S.J., Turner K.J., Davies D.R., Murray P.G., Morgan N.V., Sowter H.M., Wykoff C.C., Maher E.R., Harris A.L., Ratcliffe P.J. (2002). HIF activation identifies early lesions in VHL kidneys: Evidence for site-specific tumor suppressor function in the nephron. Cancer Cell.

[49] Perretta-Tejedor N., Jafree D.J., Long D.A. (2020). Endothelial-epithelial communication in polycystic kidney disease: Role of vascular endothelial growth factor signalling. Cell. Signal..

[50] Hanahan D., Folkman J. (1996). Patterns and emerging mechanisms of the angiogenic switch during tumorigenesis. Cell.

[51] Nagy J.A., Chang S.H., Shih S.C., Dvorak A.M., Dvorak H.F. (2010). Heterogeneity of the tumor vasculature. Semin. Thromb. Hemost..

[52] Raza A., Franklin M.J., Dudek A.Z. (2010). Pericytes and vessel maturation during tumor angiogenesis and metastasis. Am. J. Hematol..

[53] Huang J.L., Woolf A.S., Long D.A. (2013). Angiogenesis and autosomal dominant polycystic kidney disease. Pediatr. Nephrol..

[54] Bello-Reuss E., Holubec K., Rajaraman S. (2001). Angiogenesis in autosomal-dominant polycystic kidney disease. Kidney. Int..

[55] Wei W., Popov V., Walocha J.A., Wen J., Bello-Reuss E. (2006). Evidence of angiogenesis and microvascular regression in autosomal-dominant polycystic kidney disease kidneys: A corrosion cast study. Kidney Int..

[56] Huang J.L., Woolf A.S., Kolatsi-Joannou M., Baluk P., Sandford R.N., Peters D.J.M., McDonald D.M., Price K.L., Winyard P.J.D., Long D.A. (2016). Vascular endothelial growth factor C for polycystic kidney diseases. J. Am. Soc. Nephrol..

[57] Ogunlade O., Connell J.J., Huang J.L., Zhang E., Lythgoe M.F., Long D.A., Beard P. (2018). In vivo three-dimensional photoacoustic imaging of the renal vasculature in preclinical rodent models. Am. J. Physiol. Renal Physiol..

[58] Alitalo K. (2011). The lymphatic vasculature in disease. Nat. Med..

[59] Outeda P., Huso D.L., Fisher S.A., Halushka M.K., Kim H., Qian F., Germino G.G., Watnick T. (2014). Polycystin signaling is required for directed endothelial cell migration and lymphatic development. Cell Rep..

[60] Jafree D.J., Moulding D., Kolatsi-Joannou M., Perretta_Tejedor N., Price K.L., Milmoe N.J., Walsh C.L., Correra R.M., Winyard P.J., Harris P.C. (2019). Spatiotemporal dynamics and heterogeneity of renal lymphatics in mammalian development and cystic kidney disease. eLife.

[61] Carmeliet P. (2005). VEGF as a key mediator of angiogenesis in cancer. Oncology.

[62] Dimke H., Sparks M.A., Thomson B.R., Frische S., Coffman T.M., Quaggin S.E. (2015). Tubulovascular cross-talk by vascular endothelial growth factor a maintains peritubular microvasculature in kidney. J. Am. Soc. Nephrol..

[63] Song X., Di Giovanni V., He N., Wang K., Ingram A., Rosenblum N.D., Pei Y. (2009). Systems biology of autosomal dominant polycystic kidney disease (ADPKD): Computational identification of gene expression pathways and integrated regulatory networks. Hum. Mol. Genet..

[64] Ferda J., Hora M., Hes O., Ferdova E., Kreuzberg B. (2007). Assessment of the kidney tumor vascular supply by 2-phase MDCT-angiography. Eur. J. Radiol..

[65] Qian C.N., Huang D., Wondergem B., Teh B.T. (2009). Complexity of tumor vasculature in clear cell renal cell carcinoma. Cancer.

[66] Anderson H., Yap J.T., Wells P., Miller M.P., Propper D., Price P., Harris A.L. (2003). Measurement of renal tumour and normal tissue perfusion using positron emission tomography in a phase II clinical trial of razoxane. Br. J. Cancer.

[67] Schraml P., Athelogou M., Hermanns T., Huss R., Moch H. (2019). Specific immune cell and lymphatic vessel signatures identified by image analysis in renal cancer. Mod. Pathol..

[68] Moch H., Presti J.C., Sauter G., Buchholz N., Jordan P., Mihatsch M.J., Waldman F.M. (1996). Genetic aberrations detected by comparative genomic hybridization are associated with clinical outcome in renal cell carcinoma. Cancer Res..

[69] Gronwald J., Storkel S., Holtgreve-Grez H., Hadaczek P., Brinkschmidt C., Jauch A., Lubinski J., Cremer T. (1997). Comparison of DNA gains and losses in primary renal clear cell carcinomas and metastatic sites: Importance of 1q and 3p copy number changes in metastatic events. Cancer Res..

[70] Schullerus D., Herbers J., Chudek J., Kanamaru H., Kovacs G. (1997). Loss of heterozygosity at chromosomes 8p, 9p, and 14q is associated with stage and grade of non-papillary renal cell carcinomas. J. Pathol..

[71] Reutzel D., Mende M., Naumann S., Storkel S., Brenner W., Zabel B., Decker J. (2001). Genomic imbalances in 61 renal cancers from the proximal tubulus detected by comparative genomic hybridization. Cytogenet. Cell Genet..

[72] Rigola M.A., Casadevall C., Bernues M., Caballin M.R., Fuster C., Gelabert A., Egozcue J., Miro R. (2002). Analysis of kidney tumors by comparative genomic hybridization and conventional cytogenetics. Cancer Genet. Cytogenet..

[73] Alimov A., Sundelin B., Bergerheim U., Pavlenko M., Pisa P., Zetterberg A., Larsson C., Lagercrantz S. (2004). Molecular cytogenetic characterization shows higher genetic homogeneity in conventional renal cell carcinoma compared to other kidney cancers. Int. J. Oncol..

[74] Sanjmyatav J., Schubert J., Junker K. (2005). Comparative study of renal cell carcinoma by CGH, multicolor-FISH and conventional cytogenic banding analysis. Oncol. Rep..

[75] Yoshimoto T., Matsuura K., Karnan S., Tagawa H., Nakada C., Tanigawa M., Tsukamoto Y., Uchida T., Kashima K., Akizuki S. (2007). High-resolution analysis of DNA copy number alterations and gene expression in renal clear cell carcinoma. J. Pathol..

[76] Chen M., Ye Y., Yang H., Tamboli P., Matin S., Tannir N.M., Wood C.G., Gu J., Wu X. (2009). Genome-wide profiling of chromosomal alterations in renal cell carcinoma using high-density single nucleotide polymorphism arrays. Int. J. Cancer.

[77] Chen F., Zhang Y., Senbabaoglu Y., Ciriello G., Yang L., Reznik E., Shuch B., Micevic G., De Velasco G., Shinbrot E. (2016). Multilevel genomics-based taxonomy of renal cell carcinoma. Cell Rep..

[78] Clifford S.C., Walsh S., Hewson K., Green E.K., Brinke A., Green P.M., Gianelli F., Eng C., Maher E.R. (1999). Genomic organization and chromosomal localization of the human CUL2 gene and the role of von Hippel-Lindau tumor suppressor-binding protein (CUL2 and VBP1) mutation and loss in renal-cell carcinoma development. Genes Chromosomes Cancer.

[79] Buchholz B., Eckardt K.U. (2020). Role of oxygen and the HIF-pathway in polycystic kidney disease. Cell. Signal..

[80] Rosenberger C., Mandriota S., Jurgensen J.S., Wiesener M.S., Horstrup J.H., Frei U., Ratcliffe P.J., Maxwell P.H., Bachmann S., Eckardt K.U. (2002). Expression of hypoxia-inducible factor-1 and -2 in hypoxic and ischemic rat kidneys. J. Am. Soc. Nephrol..

[81] Salceda S., Caro J. (1997). Hypoxia-inducible factor 1alpha (HIF-1alpha) protein is rapidly degraded by the ubiquitin-proteasome system under normoxic conditions. Its stabilization by hypoxia depends on redox-induced changes. J. Biol. Chem..

[82] Semenza G.L. (2010). Defining the role of hypoxia-inducible factor 1 in cancer biology and therapeutics. Oncogene.

[83] Schodel J., Ratcliffe P.J. (2019). Mechanisms of hypoxia signalling: New implications for nephrology. Nat. Rev. Nephrol..

[84] Cohen H.T., McGovern F.J. (2005). Renal-cell carcinoma. N. Engl. J. Med..

[85] Guo H., German P., Bai S., Barnes S., Guo W., Qi X., Lou H., Liang J., Jonasch E., Mills G.B. (2015). The PI3K/AKT pathway in renal cell carcinoma. J. Genet. Genom..

[86] Gnarra J.R., Tory K., Weng Y., Schmidt L., Wei M.H., Li H., Latif F., Liu S., Chen F., Duh F.M. (1994). Mutations of the VHL tumour suppressor gene in renal carcinoma. Nat. Genet..

[87] Brugarolas J. (2014). Molecular genetics of clear-cell renal cell carcinoma. J. Am. Soc. Clin. Oncol..

[88] Gerlinger M., Rowan A.J., Horswell S., Larkin J., Endesfelder D., Gronroos E., Martinez P., Matthews N., Stewart A., Tarpey P. (2012). Intratumor heterogeneity and branched evolution revealed by multiregion sequencing. N. Engl. J. Med..

[89] Turajlic S., Xu H., Litchfield K., Rowan A., Horswell S., Chambers T., O’Brien T., Lopez J.I., Watkins T.B.K., Nicol D. (2018). Deterministic evolutionary trajectories influence primary tumor growth: TRACERx renal. Cell.

[90] Hakimi A.A., Chen Y.B., Wren J., Gonen M., Abdel-Wahab O., Heguy A., Liu H., Takeda S., Tickoo S.K., Reuter V.E. (2013). Clinical and pathologic impact of select chromatin-modulating tumor suppressors in clear cell renal cell carcinoma. Eur. Urol..

[91] Sato Y., Yoshizato T., Shiraishi Y., Maekawa S., Okuno Y., Kamura T., Shimamura T., Sato-Otsubo A., Nagae G., Suzuki H. (2013). Integrated molecular analysis of clear-cell renal cell carcinoma. Nat. Genet..

[92] Liao L., Testa J.R., Yang H. (2015). The roles of chromatin-remodelers and epigenetic modifiers in kidney cancer. Cancer Genet..

[93] Kim E., Zschiedrich S. (2018). Renal cell carcinoma in von Hippel-Lindau disease-From tumor genetics to novel therapeutic strategies. Front. Pediatr..

[94] Banumathy G., Cairns P. (2010). Signaling pathways in renal cell carcinoma. Cancer Biol. Ther..

[95] Linehan W.M., Srinivasan R., Schmidt L.S. (2010). The genetic basis of kidney cancer: A metabolic disease. Nat. Rev. Urol..

[96] Haase V. (2006). The VHL/HIF oxygen-sensing pathway and its relevance to kidney disease. Kidney Int..

[97] Kim S., Jee K., Kim D., Koh H., Chung J. (2001). Cyclic AMP inhibits Akt activity by blocking the membrane localization of PDK1. J. Biol. Chem..

[98] Fingar D.C., Richardson C.J., Tee A.R., Cheatham L., Tsou C., Blenis J. (2004). mTOR controls cell cycle progression through its cell growth effectors S6K1 and 4E-BP1/eukaryotic translation initiation factor 4E. Mol. Cell. Biol..

[99] Kosti A., de Araujo P.R., Li W.Q., Guardia G.D.A., Chiou J., Yi C., Ray D., Meliso F., Li Y.M., Delambre T. (2020). The RNA-binding protein SERBP1 functions as a novel oncogenic factor in glioblastoma by bridging cancer metabolism and epigenetic regulation. Genome Biol..

[100] Harris P.C., Torres V.E. (2014). Genetic mechanisms and signaling pathways in autosomal dominant polycystic kidney disease. J. Clin. Investig..

[101] Yamaguchi T., Wallace D.P., Magenheimer B.S., Hempson S.J., Grantham J.J., Calvet J.P. (2004). Calcium restriction allows cAMP cells to a cAMP-dependent growth-stimulated phenotype. J. Biol. Chem..

[102] Tao Y., Kim J., Schrier R.W., Edelstein C.L. (2005). Rapamycin markedly slows disease progression in a rat model of polycystic kidney disease. J. Am. Soc. Nephrol..

[103] Distefano G., Boca M., Rowe I., Wodarczyk C., Ma L., Piontek K.B., Germino G.G., Pandolfi P.P., Boletta A. (2009). Polycystin-1 regulates extracellular signal-regulated kinase-dependent phosphorylation of tuberin to control cell size through mTOR and its downstream effectors S6K and 4EBP1. Mol. Cell. Biol..

[104] Shillingford J.M., Piontek K.B., Germino G.G., Weimbs T. (2010). Rapamycin ameliorates PKD resulting from conditional inactivation of Pkd1. J. Am. Soc. Nephrol..

[105] Hudson C.C., Liu M., Chiang G.G., Otterness D.M., Loomis D.C., Kaper F., Giaccia A.J., Abraham R.T. (2002). Regulation of hypoxia-inducible factor 1a expression and function by the mammalian target or rapamycin. Mol. Cell. Biol..

[106] Belibi F., Zafar I., Ravichandran K., Segvic A.B., Jani A., Ljubanovic D.G., Edelstein C.L. (2011). Hypoxia-inducible factor-1a (HIF-1a) and autophagy in polycystic kidney disease (PKD). Am. J. Physiol. Ren. Physiol..

[107] Trudel M., Lanoix J., Barisoni L., Blouin M.J., Desforges M., L’Italien C., D’Agati V. (1997). C-MYC-induced apoptosis in polycystic kidney disease is Bcl-2 and p53 independent. J. Exp. Med..

[108] Couillard M., Guillaume R., Tanji N., D’Agati V., Trudel M. (2002). c-myc-induced apoptosis in polycystic kidney disease is independent of FasL/Fas interaction. Cancer Res..

[109] Wallace D.P. (2011). Cyclic AMP-mediated cyst expansion. Biochim. Biophys. Acta.

[110] Ricketts C.J., Shuch B., Vocke C.D., Metwalli A.R., Bratslavsky G., Middelton L., Yang Y., Wei M.H., Pautler S.E., Peterson J. (2012). Succinate dehydrogenase kidney cancer: An aggressive example of the Warburg effect in cancer. J. Urol..

[111] Warburg O., Posener K., Negelein E. (1924). Metabolism of the carcinoma cell. Biochem. Z..

[112] Yin C., Qie S., Sang N. (2012). Carbon source metabolism and its regulation in cancer cells. Crit. Rev. Eukaryot. Gene Expr..

[113] Furuta E., Okuda H., Kobayashi A., Watabe K. (2010). Metabolic genes in cancer: Their roles in tumor progression and clinical implications. Biochim. Biophys. Acta.

[114] Pastorekova S., Gillies R.J. (2019). The role of carbonic anhydrase IX in cancer development: Links to hypoxia, acidosis, and beyond. Cancer Metastasis Rev..

[115] Grabmaier K., Weijert M.C., Verhaegh G.W., Schalken J.A., Oosterwijk E. (2004). Strict regulation of CAIXG250/MN by HIF-1α in clear cell renal cell carcinoma. Oncogene.

[116] Drusian L., Nigro E.A., Mannella V., Pagliarini R., Pema M., Costa A.S., Benigni F., Larcher A., Chiaravalli M., Gaude E. (2018). MTORC1 upregulation leads to accumulation of the oncometabolite fumarate in a mouse model of renal cell carcinoma. Cell Rep..

[117] Lai S., Jiao B., Wang X., Xu X., Zhang M., Diao T., Zhang G. (2019). Renal cell carcinoma originating in the free wall of simple renal cyst: Two unusual case reports with literature review. Medicine.

[118] Vander Heiden M.G., Cantley L.C., Thompson C.B. (2009). Understanding the Warburg effect: The metabolic requirements of cell proliferation. Science.

[119] Rowe I., Chiaravalli M., Mannella V., Ulisse V., Quilici G., Pema M., Song X.W., Xu H., Mari S., Qian F. (2013). Defective glucose metabolism in polycystic kidney disease identifies a new therapeutic strategy. Nat. Med..

[120] Kipp K.R., Rezaei M., Lin L., Dewey E.C., Weimbs T. (2016). A mild reduction of food intake slows disease progression in an orthologous mouse model of polycystic kidney disease. Am. J. Physiol. Ren. Physiol..

[121] Torres J.A., Kruger S.L., Broderick C., Amarlkhagva T., Agrawal S., Dodam J.R., Mrug M., Lyons L.A., Weimbs T. (2019). Ketosis ameliorates renal cyst growth in polycystic kidney disease. Cell Metab..

[122] Ebert B.L., Firth J.D., Ratcliffe P.J. (1995). Hypoxia and mitochondrial inhibitors regulate expression of glucose transporter-1 via distinct cis-acting sequences. J. Biol. Chem..

[123] Kraus A., Peters D.J.M., Klanke B., Weidemann A., Willam C., Schley G., Kunzelmann K., Eckardt K.U., Buchholz B. (2018). HIF-1α promotes cyst progression in a mouse model of autosomal dominant polycystic kidney disease. Kidney Int..

[124] Bernhardt W.M., Wiesener M.S., Weidemann A., Schmitt R., Weichert W., Lechler P., Campean V., Ong A.C.M., Willam C., Gretz N. (2007). Involvement of hypoxia-inducible transcription factors in polycystic kidney disease. Am. J. Pathol..

[125] Buchholz B., Schley G., Faria D., Kroening S., Willam C., Schreiber R., Klanke B., Burzlaff N., Jantsch J., Kunzelmann K. (2014). Hypoxia-inducible factor-1alpha causes renal cyst expansion through calcium-activated chloride secretion. J. Am. Soc. Nephrol..

[126] Kocyigit I., Taheri S., Eroglu E., Sener E.F., Zararsiz G., Uzun I., Tufan E., Mehmetbeyoglu E., Bayramov K.K., Sipahioglu M.H. (2019). Systemic succinate, hypoxia-inducible Factor-1 alpha, and IL-1β gene expression in autosomal dominant polycystic kidney disease with and without hypertension. Cardiorenal Med..

[127] Schreiber R., Buchholz B., Kraus A., Schley G., Scholz J., Ousingsawat J., Kunzelmann K. (2019). Lipid peroxidation drives renal cyst growth in vitro through activation of TMEM16A. J. Am. Soc. Nephrol..

[128] Litan A., Langhans S.A. (2015). Cancer as a channelopathy: Ion channels and pumps in tumor development and progression. Front. Cell. Neurosci..

[129] Tulk B.M., Edwards J.C. (1998). NCC27, a homolog of intracellular CI-channel p64, is expressed in brush border of renal proximal tubule. Am. J. Physiol..

[130] Nesiu A., Cimpean A.M., Ceausu R.A., Adile A., Ioiart I., Porta C., Mazzanti M., Camerota T.C., Raica M. (2019). Intracellular chloride ion channel protein-1 expression in clear cell renal cell carcinoma. Cancer Genom. Proteom..

[131] Barbieri F., Wurth R., Pattarozzi A., Verduci I., Mazzola C., Cattaneo C., Tonelli M., Solari A., Bajetto A., Daga A. (2018). Inhibition of chloride intracellular channel I (CLIC1) as biguanide class-effect to impair human glioblastoma stem cell viability. Front. Pharmacol..

[132] Littler D.R., Harrop S.J., Fairlie W.D., Brown L.J., Pankhurst G.J., Pankhurst S., DeMaere M.Z., Campbell T.J., Bauskin A.R., Tonini R. (2004). The intracellular chloride ion channel protein CLIC1 undergoes a redox-controlled structural transition. J. Biol. Chem..

[133] Goodchild S.C., Howell M.W., Cordina N.M., Littler D.R., Breit S.N., Curmi P.M., Brown L.J. (2009). Oxidation promotes insertion of the CLIC1 chloride intracellular channel into the membrane. Eur. Biophys. J..

[134] Setti M., Savalli N., Osti D., Richichi C., Angelini M., Bescia P., Fornasari L., Carro M.S., Mazzanti M., Pelicci G. (2013). Functional role of CLIC1 ion channel in glioblastoma-derived stem/progenitor cells. J. Natl. Cancer Inst..

[135] Wang W., Xu X., Wang W., Shao W., Li L., Yin W., Xiu L., Mo M., Zhao J., He Q. (2011). The expression and clinical significance of CLIC1 and HSP27 in lung adenocarcinoma. Tumour Biol..

[136] Gurski L.A., Knowles L.M., Basse P.H., Maranchie J.K., Watkins S.C., Pilch J. (2014). Relocation of CLIC1 promotes tumor cell invasion and colonization of fibrin. Mol. Cancer Res..

[137] Hanaoka K., Devuyst O., Schwiebert E.M., Wilson P.D., Guggino W.B. (1996). A role for CFTR in human autosomal dominant polycystic kidney disease. Am. J. Physiol. Cell Physiol..

[138] Xia X., Wang J., Liu Y., Yue M. (2017). Lower cystic fibrosis transmembrane conductance regulator (CFTR) promotes the proliferation and migration of endometrial carcinoma. Med. Sci. Monit..

[139] Pinto C.S., Reif G.A., Nivens E., White C., Wallace D.P. (2012). Calmodulin-sensitive adenylyl cyclases mediate AVP-dependent cAMP production and Cl^-^ secretion by human autosomal dominant polycystic kidney cells. Am. J. Physiol. Ren. Physiol..

[140] Walther M.M., Patel B., Choyke P.L., Lubensky I.A., Vocke C.D., Harris C., Venzon D., Burtis W.J., Linehan W.M. (1997). Hypercalcemia in patients with metastatic renal cell carcinoma: Effect of nephrectomy and metabolic evaluation. J. Urol..

[141] Mangolini A., de Stephanis L., Aguiari G. (2016). Role of calcium in polycystic kidney disease: From signaling to pathology. World J. Nephrol..

[142] Chebib F.T., Sussman C.R., Wang X., Harris P.C., Torres V.E. (2015). Vasopressin and disruption of calcium signaling in polycystic kidney disease. Nat. Rev. Nephrol..

[143] Yamaguchi T., Hempson S.J., Reif G.A., Hedge A.M., Wallace D.P. (2006). Calcium restores a normal proliferation phenotype in human polycystic kidney disease epithelial cells. J. Am. Soc. Nephrol..

[144] Hildebrandt F., Benzing T., Katsanis N. (2011). Ciliopathies. N. Engl. J. Med..

[145] Basten S.G., Willekers S., Vermaat J.S., Slaats G.G., Voest E.E., van Diest P.J., Giles R.H. (2013). Reduced cilia frequencies in human renal cell carcinomas versus neighboring parenchymal tissue. Cilia.

[146] Kathem S.H., Mohieldin A.M., Nauli S.M. (2014). The roles of primary cilia in polycystic kidney disease. AIMS Mol. Sci..

[147] Yoder B.K. (2007). Role of primary cilia in the pathogenesis of polycystic kidney disease. J. Am. Soc. Nephrol..

[148] Frew I.J., Thoma C.R., Georgiev S., Minola A., Hitz M., Montani M., Moch H., Krek W. (2008). pVHL and PTEN tumour suppressor proteins cooperatively suppress kidney cyst formation. EMBO J..

[149] Dere R., Perkins A.L., Bawa-Khalfe T., Jonasch D., Walker C.L. (2015). Beta-catenin links von hippel-lindau to aurora kinase A and loss of primary cilia in renal cell carcinoma. J. Am. Soc. Nephrol..

[150] Kuehn E.W., Walz G., Benzing T. (2007). Von hippel-lindau: A tumor suppressor links microtubules to ciliogenesis and cancer development. Cancer Res..

[151] Pan J., Seeger-Nukpezah T., Golemis E.A. (2013). The role of the cilium in normal and abnormal cell cycles: Emphasis on renal cystic pathologies. Cell. Mol. Life Sci..

[152] Pugacheva E.N., Jablonski S.A., Hartman T.R., Henske E.P., Golemis E.A. (2007). HEF1-dependent aurora A activation induces disassembly of the primary cilium. Cell.

[153] Esteban M.A., Harten S.K., Tran M.G., Maxwell P.H. (2006). Formation of primary cilia in the renal epithelium is regulated by the von Hippel-Lindau tumor suppressor protein. J. Am. Soc. Nephrol..

[154] Plotnikova O.V., Pugacheva E.N., Golemis E.A. (2011). Aurora A kinase activity influences calcium signaling in kidney cells. J. Am. Soc. Nephrol..

[155] Nikonova A.S., Plotnikova O.V., Serzhanova V., Efimov A., Bogush I., Cai K.Q., Hensley H.H., Egleston B.L., Klein-Szanto A., Seeger-Nukpezah T. (2014). Nedd9 restrains renal cystogenesis in Pkd1^−/−^ mice. Proc. Natl. Acad. Sci. USA.

[156] Ma M., Tian X., Igarashi P., Pazour G.J., Somlo S. (2013). Loss of cilia suppresses cyst growth in genetic models of autosomal dominant polycystic kidney disease. Nat. Genet..

[157] Nikonova A.S., Deneka A.Y., Eckman L., Kopp M.C., Hensley H.H., Egleston B.L., Golemis E.A. (2015). Opposing effects of inhibitors of Aurora-A and EGFR in autosomal-dominant polycystic kidney disease. Front. Oncol..

[158] Lee J.E., Gleeson J.G. (2011). A systems-biology approach to understanding the ciliopathy disorders. Genome Med..

[159] Pazour G.J., San Agustin J.T., Follit J.A., Rosenbaum J.L., Witman G.B. (2002). Polycystin-2 localizes to kidney cilia and the ciliary level is elevated in orpk mice with polycystic kidney disease. Curr. Biol..

[160] Yoder B.K., Hou X., Guay-Woodford L.M. (2002). The polycystic kidney disease proteins, polycystin-1, polycystin-2, polaris, and cystin, are co-localized in renal cilia. J. Am. Soc. Nephrol..

[161] Moyer J.H., Lee-Tischler M.J., Kwon H.Y., Schrick J.J., Avner E.D., Sweeney W.E., Godfrey V.L., Cacheiro N.L., Wilkinson J.E., Woychik R.P. (1994). Candidate gene associated with a mutation causing recessive polycystic kidney disease in mice. Science.

[162] Pazour G.J., Baker S.A., Deane J.A., Cole D.G., Dickert B.L., Rosenbaum J.L., Witman G.B., Besharse J.C. (2002). The intraflagellar transport protein, IFT88, is essential for vertebrate photoreceptor assembly and maintenance. J. Cell Biol..

[163] Kramer-Zucker A.G., Olale F., Haycraft C.J., Yoder B.K., Schier A.F., Drummond I.A. (2005). Cilia-driven fluid flow in the zebrafish pronephros, brain and Kupffer’s vesicle is required for normal organogenesis. Development.

[164] Obara T., Mangos S., Liu Y., Zhao J., Wiessner S., Kramer-Zucker A.G., Olale F., Schier A.F., Drummond I.A. (2006). Polycystin-2 immunolocalization and function in zebrafish. J. Am. Soc. Nephrol..

[165] Mangos S., Lam P.Y., Zhao A., Liu Y., Mudumana S., Vasilyev A., Liu A., Drummond I.A. (2010). The ADPKD genes pkd1a/b and pkd2 regulate extracellular matrix formation. Dis. Model Mech..

[166] Gamberi C., Hipfner D.R., Trudel M., Lubell W.D. (2017). Bicaudal C mutation causes myc and TOR pathway up-regulation and polycystic kidney disease-like phenotypes in drosophila. PLoS Genet..

[167] Torres V.E., Chapman A.B., Devuyst O., Gansevoort R.T., Perrone R.D., Koch G., Ouyang J., McQuade R.D., Blais J.D., Czerwiec F.S. (2017). Tolvaptan in later-stage autosomal dominant polycystic kidney disease. N. Engl. J. Med..

[168] Sinha S., Dwivedi N., Tao S., Jamadar A., Kakade V.R., O’Neil M., Weiss R.H., Enders J., Calvet J.P., Thomas S.M. (2020). Targeting the vasopressin type-2 receptor for renal cell carcinoma therapy. Oncogene.

[169] Sherpa R.T., Mohieldin A.M., Pala R., Wachten D., Ostrom R.S., Nauli S.M. (2019). Sensory primary cilium is a responsive cAMP microdomain in renal epithelia. Sci. Rep..

[170] Dasgupta P., Kulkarni P., Majid S., Shahryari V., Hashimoto Y., Bhat N.S., Shiina M., Deng G., Saini S., Tabatabai Z.L. (2018). MicroRNA-203 inhibits long noncoding RNA hotair and regulates tumorigenesis through epithelial-to-mesenchymal transition pathway in renal cell carcinoma. Mol. Cancer Ther..

[171] Ren Y., Huang W., Weng G., Cui P., Liang H., Li Y. (2019). LncRNA PVT1 promotes proliferation, invasion and epithelial–mesenchymal transition of renal cell carcinoma cells through downregulation of miR-16-5p. OncoTargets Ther..

[172] Xie W., Chen Q., Liu X.I.N., Ma M., Yang X., Gong B., Sun T., Chen J. (2019). Silencing of the long non-coding RNA GHET1 inhibits cell proliferation and migration of renal cell carcinoma through epithelial-mesenchymal transition. Oncol. Lett..

[173] Joosten S.C., Smits K.M., Aarts M.J., Melotte V., Koch A., Tjan-Heijnen V.C., Van Engeland M. (2018). Epigenetics in renal cell cancer: Mechanisms and clinical applications. Nat. Rev. Urol..

[174] Patel V., Hajarnis S., Williams D., Hunter R., Huynh D., Igarashi P. (2012). MicroRNAs regulate renal tubule maturation through modulation of Pkd1. J. Am. Soc. Nephrol..

[175] Gilyazova I.R., Klimentova E.A., Bulygin K.V., Izmailov A.A., Bermisheva M.A., Galimova E.F., Safiullin R.I., Galimov S.N., Pavlov V.N., Khusnutdinova E.K. (2020). MicroRNA-200 family expression analysis in metastatic clear cell renal cell carcinoma patients. Cancer Gene Ther..

[176] Jiang J., Yi B., Ding S., Sun J., Cao W., Liu M. (2016). Demethylation drug 5-AZA-2′-deoxycytidine-induced upregulation of miR-200c inhibits the migration, invasion and epithelial-mesenchymal transition of clear cell renal cell carcinoma in vitro. Oncol. Lett..

[177] Hajarnis S., Lakhia R., Yheskel M., Williams D., Sorourian M., Liu X., Aboudehen K., Zhang S., Kersjes K., Galasso R. (2017). MicroRNA-17 family promotes polycystic kidney disease progression through modulation of mitochondrial metabolism. Nat. Commun..

[178] Chow T.F., Mankaruos M., Scorilas A., Youssef Y., Girgis A., Mossad S., Metias S., Rofael Y., Honey R.J., Stewart R. (2010). The miR-17-92 cluster is over expressed in and has an oncogenic effect on renal cell carcinoma. J. Urol..

[179] Faragalla H., Youssef Y.M., Scorilas A., Khalil B., White N.M.A., Mejia-Guerrero S., Khella H., Jewett M.A.S., Evans A., Lichner Z. (2012). The clinical utility of miR-21 as a diagnostic and prognostic marker for renal cell carcinoma. J. Mol. Diagn..

[180] Lakhia R., Hajarnis S., Williams D., Aboudehen K., Yheskel M., Xing C., Hatley M.E., Torres V.E., Wallace D.P., Patel V. (2016). MicroRNA-21 aggravates cyst growth in a model of polycystic kidney disease. J. Am. Soc. Nephrol..

[181] Huang T., Wang X., Yang X., Ji J., Wang Q., Yue X., Dong Z. (2018). Long non-coding RNA DUXAP8 enhances renal cell carcinoma progression via downregulating miR-126. Med. Sci. Monit..

[182] Cao Y., Xu R., Xu X., Zhou Y., Cui L., He X. (2016). Downregulation of lncRNA CASC2 by microRNA-21 increases the proliferation and migration of renal cell carcinoma cells. Mol. Med. Rep..

[183] Aboudehen K., Farahani S., Kanchwala M., Chan S.C., Avdulov S., Mickelson A., Lee D., Gearhart M.D., Patel V., Xing C. (2018). Long noncoding RNA *Hoxb3os* is dysregulated in autosomal dominant polycystic kidney disease and regulates mTOR signaling. J. Biol. Chem..

[184] Lam J.K.W., Chow M.Y.T., Zhang Y., Leung S.W.S. (2015). siRNA versus miRNA as therapeutics for gene silencing. Mol. Ther. Nucleic Acids.

[185] Mytsyk Y., Dosenko V., Skrzypczyk M.A., Borys Y., Diychuk Y., Kucher A., Kowalskyy V., Pasichnyk S., Mytsyk O., Manyuk L. (2018). Potential clinical applications of microRNAs as biomarkers for renal cell Carcinoma. Cent. Eur. J. Urol..

[186] Ma H., Pan J.S., Jin L.X., Wu J., Ren Y.D., Chen P., Xiao C., Han J. (2016). MicroRNA-17~92 inhibits colorectal cancer progression by targeting angiogenesis. Cancer Lett..

[187] Kang H.M., Ahn S.H., Choi P., Ko Y.A., Han S.H., Chinga F., Park A.S.D., Tao J., Sharma K., Pullman J. (2015). Defective fatty acid oxidation in renal tubular epithelial cells has a key role in kidney fibrosis development. Nat. Med..

[188] Lakhia R., Yheskel M., Flaten A., Quittner-Strom E.B., Holland W.L., Patel V. (2018). PPARα agonist fenofibrate enhances fatty acid β-oxidation and attenuates polycystic kidney and liver disease in mice. Am. J. Physiol. Renal Physiol..

[189] Noureddine L., Hajarnis S., Patel V. (2013). MicroRNAs and polycystic kidney disease. Drug Discov. Today Dis. Models.

[190] Zhang G.M., Luo L., Ding X.M., Dong D.H., Li B., Ma X.C., Sun L.J. (2016). MicroRNA-126 inhibits tumor cell invasion and metastasis by downregulating ROCK1 in renal cell carcinoma. Mol. Med. Rep..

[191] Pourkarimi E., Greiss S., Gartner A. (2012). Evidence that CED-9/Bcl2 and CED-4/Apaf-1 localization is not consistent with the current model for C. elegans apoptosis induction. Cell Death Differ..

[192] Flybase. flybase.org.

[193] Schor I.E., Bussotti G., Maleš M., Forneris M., Viales R.R., Enright A.J., Furlong E.E.M. (2018). Non-coding RNA expression, function, and variation during *Drosophila* embryogenesis. Curr. Biol..

[194] Li K., Tian Y., Yuan Y., Fan X., Yang M., He Z., Yang D. (2019). Insights into the functions of LncRNAs in *Drosophila*. Int. J. Mol. Sci..

[195] Millet-Boureima C., Porras Marroquin J., Gamberi C. (2018). Modeling renal disease “On the fly”. BioMed Res. Int..

[196] Wang J., Kean L., Yang J., Allan A.K., Davies S.A., Herzyk P., Dow J.A.T. (2004). Function-informed transcriptome analysis of *Drosophila* renal tubule. Genome Biol..

[197] Chien S., Reiter L.T., Bier E., Gribskov M. (2002). Homophila: Human disease gene cognates in *Drosophila*. Nucleic Acids Res..

[198] Chicoine J., Benoit P., Gamberi C., Paliouras M., Simonelig M., Lasko P. (2007). Bicaudal-C recruits CCR4-NOT deadenylase to target mRNAs and regulates oogenesis, cytoskeletal organization, and its own expression. Dev. Cell.

[199] Gamberi C., Lasko P. (2012). The Bic-C family of developmental translational regulators. Comp. Funct. Genom..

[200] Millet-Boureima C., Chingle R., Lubell W.D., Gamberi C. (2019). Cyst reduction in a polycystic kidney disease *Drosophila* model using Smac mimics. Biomedicines.

[201] Millet-Boureima C., Selber-Hnatiw S., Gamberi C. (2020). Drug discovery and chemical probing in *Drosophila*. Genome.

[202] Li L., Yang Y., Xue L. (2010). Regulatory functions of Pax gene family in *Drosophila* development. Yi Chuan.

[203] Li C.G., Chantry A., Stayner C., Horsfield J., Eccles M.R. (2018). SMAD proteins directly suppress PAX2 transcription downstream of transforming growth factor-beta 1 (TGF-β1) signalling in renal cell carcinoma. Oncotarget.

[204] Zhang H., Stallock J.P., Ng J.C., Reinhard C., Neufeld T.P. (2000). Regulation of cellular growth by the *Drosophila* target of rapamycin *dTOR*. Genes Dev..

[205] Gallant P. (2013). Myc function in *Drosophila*. Cold Spring Harb. Perspect. Med..

[206] Trumpp A., Refaeli Y., Oskarsson T., Gasser S., Murphy M., Martin G.R., Bishop J.M. (2001). c-Myc regulates mammalian body size by controlling cell number but not cell size. Nature.

[207] Gao X., Neufeld T.P., Pan D. (2000). *Drosophila* PTEN regulates cell growth and proliferation through PI3K-dependent and -independent pathways. Dev. Biol..

[208] Bader H.L., Hsu T. (2012). Systemic VHL gene functions and the VHL disease. FEBS Lett..

[209] Adryan B., Decker H.J., Papas T.S., Hsu T. (2000). Tracheal development and the von Hippel-Lindau tumor suppressor homolog in *Drosophila*. Oncogene.

[210] Hsouna A., Nallamothu G., Kose N., Guinea M., Dammai V., Hsu T. (2010). *Drosophila* von Hippel-Lindau tumor suppressor gene function in epithelial tubule morphogenesis. Mol. Cell. Biol..

[211] Hsu T. (2012). Complex cellular functions of the von Hippel-Lindau tumor suppressor gene: Insights from model organisms. Oncogene.

[212] Ghabrial A., Luschnig S., Metzstein M.M., Krasnow M.A. (2003). Branching morphogenesis of the *Drosophila* tracheal system. Annu. Rev. Cell Dev. Biol..

[213] Dammai V., Adryan B., Lavenburg K.R., Hsu T. (2003). *Drosophila* awd, the homolog of human nm23, regulates FGF receptor levels and functions synergistically with *shi*/*dynamin* during tracheal development. Genes Dev..

[214] Duchi S., Fagnocchi L., Cavaliere V., Hsouna A., Gargiulo G., Hsu T. (2010). *Drosophila* VHL tumor-suppressor gene regulates epithelial morphogenesis by promoting microtubule and aPKC stability. Development.

[215] Ignesti M., Andrenacci D., Fischer B., Cavaliere V., Gargiulo G. (2019). Comparative expression profiling of wild type *Drosophila* Malpighian tubules and von Hippel-Lindau haploinsufficient mutant. Front. Physiol..

[216] Peri S., Caretti E., Tricarico R., Devarajan K., Cheung M., Sementino E., Menges C.W., Nicolas E., Vanderveer L.A., Howard S. (2017). Haploinsufficiency in tumor predisposition syndromes: Altered genomic transcription in morphologically normal cells heterozygous for VHL or TSC mutation. Oncotarget.

[217] Peri S., Devarajan K., Yang D.H., Knudson A.G., Balachandran S. (2013). Meta-analysis identifies NF-B as a therapeutic target in renal cancer. PLoS ONE.

[218] Bangi E., Ang C., Smibert P., Uzilov A.V., Teague A.G., Antipin Y., Chen R., Hecht C., Gruszczynski N., Yon W.J. (2019). A personalized platform identifies trametinib plus zoledronate for a patient with KRAS-mutant metastatic colorectal cancer. Sci. Adv..

[219] Mortimer N.T., Moberg K.H. (2009). Regulation of *Drosophila* embryonic tracheogenesis by dVHL and hypoxia. Dev. Biol..

[220] Centanin L., Dekanty A., Romero N., Irisarri M., Gorr T.A., Wappner P. (2008). Cell autonomy of HIF effects in *Drosophila*: Tracheal cells sense hypoxia and induce terminal branch sprouting. Dev. Cell.

[221] Li Y., Padmanabha D., Gentile L.B., Dumur C., Beckstead R.B., Baker K.D. (2013). HIF- and non-HIF-regulated hypoxic responses require the estrogen-related in *Drosophila melanogaster*. PLoS Genet..

[222] Sonnenfeld M., Ward M., Nystrom G., Mosher J., Stahl S., Crews S. (1997). The *Drosophila tango* gene encodes a bHLH-PAS protein that is orthologous to mammalian Arnt and controls CNS midline and tracheal development. Development.

[223] Mukherjee T., Kim W.S., Mandal L., Banerjee U. (2011). Interaction between Notch and Hif-in development and survival of *Drosophila* blood cells. Science.

[224] Cho N.K., Keyes L., Johnson E., Heller J., Ryner L., Karim F., Krasnow M.A. (2002). Developmental control of blood cell migration by the *Drosophila* VEGF pathway. Cell.

[225] Igaki T. (2009). Correcting developmental errors by apoptosis: Lessons from *Drosophila* JNK signaling. Apoptosis.

[226] Ratheesh A., Belyaeva V., Siekhaus D.E. (2015). *Drosophila* immune cell migration and adhesion during embryonic development and larval immune responses. Curr. Opin. Cell Biol..

[227] Read R.D., Cavenee W.K., Furnari F.B., Thomas J.B. (2009). A *Drosophila* model for EGFR-Ras and PI3K-dependent human glioma. PLoS Genet..

[228] Buchon N., Broderick N.A., Kuraishi T., Lemaitre B. (2010). *Drosophila* EGFR pathway coordinates stem cell proliferation and gut remodeling following infection. BMC Biol..

[229] Volkenhoff A., Hirrlinger J., Kappel J.M., Klambt C., Schirmeier S. (2018). Live imaging using a FRET glucose sensor reveals glucose delivery to all cell types in the *Drosophila* brain. J. Insect Physiol..

[230] Mohrmann L., Langenberg K., Krijgsveld J., Kal A.J., Heck A.J.R., Verrijzer C.P. (2004). Differential targeting of two distinct SWI/SNF-related *Drosophila* chromatin-remodeling complexes. Mol. Cell Biol..

[231] Scheuermann J.C., de Ayala Alonso A.G., Oktaba K., Ly-Hartig N., McGinty R.K., Fraterman S., Wilm M., Muir T.W., Muller J. (2010). Histone H2A deubiquitinase activity of the Polycomb repressive complex PR-DUB. Nature.

[232] Foglizzo M., Middleton A.J., Burgess A.E., Crowther J.M., Dobson R.C.J., Murphy J.M., Day C.L., Mace P.D. (2018). A bidentate Polycomb Repressive-Deubiquitinase complex is required for efficient activity on nucleosomes. Nat. Commun..

[233] Wang C.I., Alekseyenko A.A., LeRoy G., Elia A.E.H., Gorchakov A.A., Britton L.M.P., Elledge S.J., Kharchenko P.V., Garcia B.A., Kuroda M.I. (2013). Chromatin proteins captured by ChIP-mass spectrometry are linked to dosage compensation in *Drosophila*. Nat. Struct. Mol. Biol..

[234] Oldham S., Montagne J., Radimerski T., Thomas G., Hafen E. (2000). Genetic and biochemical characterization of dTOR, the *Drosophila* homolog of the target of rapamycin. Genes Dev..

[235] Johnston L.A., Prober D.A., Edgar B.A., Eisenman R.N., Gallant P. (1999). *Drosophila myc* regulates cellular growth during development. Cell.

[236] Goberdhan D.C.I., Paricio N., Goodman E.C., Miodzik M., Wilson C. (1999). *Drosophila* tumor suppressor PTEN controls cell size and number by antagonizing the Chico/PI3-kinase signaling pathway. Genes Dev..

[237] Huang H., Potter C.J., Tao W., Li D.M., Brogiolo W., Hafen E., Sun H., Xu T. (1999). PTEN affects cell size, cell proliferation and apoptosis during *Drosophila* eye development. Development.

[238] Yamada Y., Davis K.D., Coffman C.R. (2008). Programmed cell death of primordial germ cells in *Drosophila* is regulated by p53 and the Outsiders monocarboxylate transporter. Development.

[239] Fan Y., Lee T.V., Xu D., Chen Z., Lamblin A.F., Steller H., Bergmann A. (2010). Dual roles of *Drosophila* p53 in cell death and cell differentiation. Cell Death Differ..

[240] Shlevkov E., Morata G. (2012). A dp53/JNK-dependent feedback amplification loop is essential for the apoptotic response to stress in *Drosophila*. Cell Death Differ..

[241] Rougeot J., Renard M., Randsholt N.B., Peronnet F., Mouchel-Vielh E. (2013). The Elongin complex antagonizes the chromatin factor corto for vein versus intervein cell identity in *Drosophila* wings. PLoS ONE.

[242] Minguet J., Smith K.H., Bramlage C.P., Bramlage P. (2015). Targeted therapies for treatment of renal cell carcinoma: Recent advances and future perspectives. Cancer Chemother. Pharmacol..

[243] Rini B.I. (2008). Temsirolimus, an inhibitor of mammalian target of rapamycin. Clin. Cancer Res..

[244] Voss M.H., Hakimi A.A., Pham C.G., Brannon A.R., Chen Y.B., Cunha L.F., Akin O., Liu H., Takeda S., Scott S.N. (2014). Tumor genetic analyses of patients with metastatic renal cell carcinoma and extended benefit from mTOR inhibitor therapy. Clin. Cancer Res..

[245] Kwiatkowski D.J., Choueiri T.K., Fay A.P., Rini B.I., Thorner A.R., de Velasco G., Tyburczy M.E., Hamieh L., Albiges L., Agarwal N. (2016). Mutations in TSC1, TSC2, and MTOR are associated with response to rapalogs in patients with metastatic renal cell carcinoma. Clin. Cancer Res..

[246] Lim S.M., Park H.S., Kim S., Kim S., Ali S.M., Greenbowe J.R., Yang I.S., Kwon N.J., Lee J.L., Ryu M.H. (2016). Next-generation sequencing reveals somatic mutations that confer exceptional response to everolimus. Oncotarget.

[247] Shillingford J.M., Murcia N.S., Larson C.H., Low S.H., Hedgepeth R., Brown N., Flask C.A., Novick A.C., Goldfarb D.A., Kramer-Zucker A. (2006). The mTOR pathway is regulated by polycystin-1, and its inhibition reverses renal cystogenesis in polycystic kidney disease. Proc. Natl. Acad. Sci. USA.

[248] Wahl P.R., Serra A.L., Le Hir M., Molle K.D., Hall M.N., Wuthrich R.P. (2006). Inhibition of mTOR with sirolimus slows disease progression in Han:SPRD rats with autosomal dominant polycystic kidney disease (ADPKD). Nephrol. Dial. Transplant..

[249] Wu M., Wahl P.R., Le Hir M., Wackerle-Men Y., Wuthrich R.P., Serra A.L. (2007). Everolimus retards cyst growth and preserves kidney function in a rodent model for polycystic kidney disease. Kidney Blood Press. Res..

[250] Torres V.E., Boletta A., Chapman A., Gattone V., Pei Y., Qian Q., Wallace D.P., Weimbs T., Wuthrich R.P. (2010). Prospects for mTOR inhibitor use in patients with polycystic kidney disease and hamartomatous diseases. Clin. J. Am. Soc. Nephrol..

[251] Zaza G., Tomei P., Ria P., Granata S., Boschiero L., Lupo A. (2013). Systemic and nonrenal adverse effects occurring in renal transplant patients treated with mTOR inhibitors. Clin. Dev. Immunol..

[252] Zaza G., Granata S., Tomei P., Masola V., Gambaro G., Lupo A. (2014). mTOR inhibitors and renal allograft: Yin and Yang. J. Nephrol..

[253] Rizzo M., Porta C. (2017). Sunitinib in the treatment of renal cell carcinoma: An update on recent evidence. Ther. Adv. Urol..

[254] Facchini G., Rossetti S., Berretta M., Cavaliere C., Scagliarini S., Vitale M.G., Ciccarese C., Di Lorenzo G., Palesandro E., Conteduca V. (2019). Second line therapy with axitinib after only prior sunitinib in metastatic renal cell cancer: Italian multicenter real world SAX study final results. J. Transl. Med..

[255] Rini B.I. (2006). Sorafenib. Exp. Opin. Pharmacother..

[256] Jäger D., Ma J.H., Mardiak J., Ye D.W., Korbenfeld E., Zemanova M., Ahn H., Guo J., Leonhartsberger N., Stauch K. (2015). Sorafenib treatment of advanced renal cell carcinoma patients in daily practice: The large international PREDICT study. Clin. Genitourin. Cancer.

[257] Roviello G., Corona S.P., Bozza G., Aieta M., Generali D., Rodriquenz M.G., Mileo A.M., Imperatori M., Ianza A., Conca R. (2018). Lenvatinib for the treatment of renal cell carcinoma. Expert Opin. Investig. Drugs.

[258] Motzer R.J., Hutson T.E., Glen H., Michaelson M.D., Molina A., Eisen T., Jassem J., Zolnierek J., Maroto J.P., Mellado B. (2015). Lenvatinib, everolimus, and the combination in patients with metastatic renal cell carcinoma: A randomised, phase 2, open-label, multicentre trial. Lancet Oncol..

[259] De Lisi D., De Giorgi U., Lolli C., Schepisi G., Conteduca V., Menna C., Tonini G., Santini D., Farolfi A. (2018). Lenvatinib in the management of metastatic renal cell carcinoma: A promising combination therapy?. Expert Opin. Drug Metab. Toxicol..

[260] Semrad T.J., Groshen S., Luo C., Pal S., Vaishampayan U., Joshi M., Quinn D.I., Mack P.C., Gandara D.R., Lara P.N. (2019). Randomized phase 2 study of Trebananib (AMG 386) with or without continued anti-vascular endothelial growth factor therapy in patients with renal cell carcinoma who have progressed on Bevacizumab, Pazopanib, Sorafenib, or Sunitinib—Results of NCI/CTEP protocol 9048. Kidney Cancer.

[261] Wang X., Solban N., Khanna P., Callea M., Song J., Alsop D.C., Pearsall R.S., Atkins M.B., Mier J.W., Signoretti S. (2016). Inhibition of ALK1 signaling with dalantercept combined with VEGFR TKI leads to tumor stasis in renal cell carcinoma. Oncotarget.

[262] Voss M.H., Bhatt R.S., Vogelzang N.J., Fishman M., Alter R.S., Rini B.I., Beck J.T., Joshi M., Hauke R., Atkins M.B. (2019). A phase 2, randomized trial evaluating the combination of dalantercept plus axitinib in patients with advanced clear cell renal cell carcinoma. Cancer.

[263] Massari F., Santoni M., Ciccarese C., Santini D., Alfieri S., Martignoni G., Brunelli M., Piva F., Berardi R., Montironi R. (2015). PD-1 blockade therapy in renal cell carcinoma: Current studies and future promises. Cancer Treat. Rev..

[264] Pardoll D.M. (2012). Blockade of immune checkpoints in cancer immunotherapy. Nat. Rev. Cancer.

[265] Thompson R.H., Dong H., Kwon E.D. (2007). Implications of B7-H1 expression in clear cell carcinoma of the kidney for prognostication and therapy. Clin. Cancer Res..

[266] Callea M., Genega E.M., Gupta M., Cheng S., Fay A.P., Song J., Carvo I., Bhatt R.S., McDermott D.F., Atkins M.B. (2014). PD-L1 expression in primary clear cell renal cell carcinomas (ccRCCs) and their metastases. J. Clin. Oncol..

[267] Jilaveanu L.B., Shuch B., Zito C.R., Parisi F., Barr M., Kluger Y., Chen L., Kluger H.M. (2014). PD-L1 expression in clear cell renal cell carcinoma: An analysis of nephrectomy and sites of metastases. J. Cancer.

[268] Kammerer-Jacquet S.F., Deleuze A., Saout J., Mathieu R., Laguerre B., Verhoest G., Dugay F., Belaud-Rotureau M.A., Bensalah K., Rioux-Leclercq N. (2019). Targeting the PD-1/PD-L1 pathway in renal cell carcinoma. Int. J. Mol. Sci..

[269] Deleuze A., Saout J., Dugay F., Peyronnet B., Mathieu R., Verhoest G., Bensalah K., Crouzet L., Laguerre B., Belaud-Rotureau M.A. (2020). Immunotherapy in renal cell carcinoma: The future is now. Int. J. Mol. Sci..

[270] Chen D.J., Huerta S. (2009). Smac mimetics as new cancer therapeutics. Anticancer Drugs.

[271] Van Gurp M., Festjens N., van Loo G., Saelens X., Vandenabeele P. (2003). Mitochondrial intermembrane proteins in cell death. Biochem. Biophys. Res. Commun..

[272] Srinivasula S.M., Datta P., Fan X.J., Fernandes-Alnemri T., Huang Z., Alnemri E.S. (2000). Molecular determinants of the caspase-promoting activity of Smac/DIABLO and its role in the death receptor pathway. J. Biol. Chem..

[273] Srinivasula S.M., Hegde R., Saleh A., Datta P., Shiozaki E., Chai J., Lee R.A., Robbins P.D., Fernandes-Alnemri T., Shi Y. (2001). A conserved XIAP-interaction motif in caspase-9 and Smac/DIABLO regulates caspase activity and apoptosis. Nature.

[274] Lalaoui N., Vaux D.L. (2018). Recent advances in understanding inhibitor of apoptosis proteins. F1000Research.

[275] Cong H., Xu L., Wu Y., Qu Z., Bian T., Zhang W., Xing C., Zhuang C. (2019). Inhibitor of apoptosis protein (IAP) antagonists in anticancer discovery: Current status and perspectives. J. Med. Chem..

[276] Mizutani Y., Nakanishi H., Yamamoto K., Li Y.N., Matsubara H., Mikami K., Okihara K., Kawauchi A., Bonavida B., Miki T. (2005). Downregulation of Smac/DIABLO expression in renal cell carcinoma and its prognostic significance. J. Clin. Oncol..

[277] Yan Y., Mahotka C., Heikaus S., Shibata T., Wethkamp N., Liebmann J., Suschek C.V., Guo Y., Gabbert H.E., Gerharz C.D. (2004). Disturbed balance of expression between XIAP and Smac/DIABLO during tumour progression in renal cell carcinomas. Br. J. Cancer.

[278] Kempkensteffen C., Hinz S., Christoph F., Krause H., Magheli A., Schrader M., Schostak M., Miller K., Weikert S. (2008). Expression levels of the mitochondrial IAP antagonists Smac/DIABLO and Omi/HtrA2 in clear-cell renal cell carcinomas and their prognostic value. J. Cancer Res. Clin. Oncol..

[279] Millet-Boureima C. (2019). Cyst Reduction in a First-in-Kind *Drosophila* Model of Polycystic Kidney Disease. Master’s Thesis.

[280] Fan L.X., Zhou X., Sweeney W.E., Wallace D.P., Avner E.D., Grantham J.J., Li X. (2013). Smac-mimetic-induced epithelial cell death reduces the growth of renal cysts. J. Am. Soc. Nephrol..

[281] Li X., Magenheimer B.S., Xia S., Johnson T., Wallace D.P., Calvet J.P., Li R. (2008). A tumor necrosis factor-alpha-mediated pathway promoting autosomal dominant polycystic kidney disease. Nat. Med..

[282] Igaki T., Kanda H., Yamamoto-Gotom Y., Kanuka H., Kuranaga E., Aigaki T., Miura M. (2002). Eiger, a TNF superfamily ligand that triggers the *Drosophila* JNK pathway. EMBO J..

[283] Kanda H., Igaki T., Kanuka H., Yagi T., Miura M. (2002). Wengen, a member of the *Drosophila* tumor necrosis factor receptor superfamily, is required for Eiger signaling. J. Biol. Chem..

[284] Moreno E., Yan M., Basler K. (2002). Evolution of TNF signaling mechanisms: JNK-dependent apoptosis triggered by Eiger, the *Drosophila* homolog of the TNF superfamily. Curr. Biol..

[285] Andersen D.S., Colombani J., Palmerini V., Chakrabandhu K., Boone E., Rothlisberger M., Toggweiler J., Basler J., Mapelli M., Hueber A.O. (2015). The *Drosophila* TNF receptor Grindelwald couple loss of cell polarity and neoplastic growth. Nature.

[286] Dosquet C., Schaetz A., Faucher C., Lepage C., Wautier J.L., Richard F., Cabane J. (1994). Tumor necrosis factor-a, interleukin-1b and interleukin-6 in patients with renal cell carcinoma. Eur. J. Cancer.

[287] Yoshida N., Ikemoto S., Narita K., Sugimura K., Wada S., Yasumoto R., Kishimoto T., Nakatani T. (2002). Interleukin-6, tumor necrosis factor alpha and interleukin-1beta in patients with renal cell carcinoma. Br. J. Cancer.

[288] Mikami S., Mizuno R., Kosaka T., Saya H., Oya M., Okada Y. (2015). Expression of TNF- and CD44 is implicated in poor prognosis, cancer cell invasion, metastasis and resistance to the sunitinib treatment in clear cell renal cell carcinomas. Int. J. Cancer.

[289] Chuang M.J., Sun K.H., Tang S.J., Deng M.W., Wu Y.H., Sung J.S., Cha T.L., Sun G.H. (2008). Tumor-derived tumor necrosis factor-alpha promotes progression and epithelial-mesenchymal transition in renal cell carcinoma cells. Cancer Sci..

[290] Cipolla-Neto J., Gaspar do Amaral F. (2018). Melatonin as a hormone: New physiological and clinical insights. Endocr. Rev..

[291] Zhao L., Hu C., Zhang P., Jiang H., Chen J. (2019). Melatonin preconditioning is an effective strategy for mesenchymal stem cell-based therapy for kidney disease. J. Cell Mol. Med..

[292] Dilman V.M., Anisimov V.N., Ostroumova M.N., Khavinson V.K., Morozov V.G. (1979). Increase in lifespan of rats following polypeptide pineal extract treatment. Exp. Pathol..

[293] Anisimov V.N., Mylnikov S.V., Oparina T.I., Khavinson V.K. (1997). Effect of melatonin and pineal peptide preparation epithalamin on life span and free radical oxidation in *Drosophila melanogaster*. Mech. Ageing Dev..

[294] Anisimov V.N., Zavarina N.Y., Zabezhinski M.A., Popovich I.G., Zimina O.A., Shtylick A.V., Arutjunyan A.V., Oparina T.I., Prokopenko V.M., Mikhalski A.I. (2001). Melatonin increases both life span and tumor incidence in female CBA mice. J. Gerontol. A Biol. Sci. Med. Sci..

[295] Anisimov V.N., Popovich I.G., Zabezhinski M.A., Anisimov S.V., Venushkin G.M., Vinogradova I.A. (2006). Melatonin as antioxidant, geroprotector and anticarcinogen. Biochim. Biophys. Acta.

[296] Su S., Hsieh M.J., Yang W.E., Chung W.H., Reiter R.J., Yang S.F. (2017). Cancer metastasis: Mechanisms of inhibition by melatonin. J. Pineal Res..

[297] Pourhanifeh M.H., Sharifi M., Reiter R.J., Davoodabadi A., Asemi Z. (2019). Melatonin and non-small cell lung cancer: New insights into signaling pathways. Cancer Cell Int..

[298] Reiter R.J. (2004). Mechanisms of cancer inhibition by melatonin. J. Pineal Res..

[299] Li Y., Li S., Zhou Y., Meng X., Zhang J.J., Xu D.P., Li H.B. (2017). Melatonin for the prevention and treatment of cancer. Oncotarget.

[300] Reiter R.J., Rosales-Corral S.A., Tan D.X., Acuna-Castroviejo D., Qin L., Yang S.F., Xu K. (2017). Melatonin, a full service and anti-cancer agent: Inhibition of initiation, progression and metastasis. Int. J. Mol. Sci..

[301] Chen H.H., Lin K.C., Wallace C.G., Chen Y.T., Yang C.C., Leu S., Chen Y.C., Sun C.K., Tsai T.H., Chen Y.L. (2014). Additional benefit of combined therapy with melatonin and apoptotic adipose-derived mesenchymal stem cell against sepsis-induced kidney injury. J. Pineal Res..

[302] Saberi K., Pasbakhsh P., Omidi A., Borhani-Haghighi M., Nekoonam S., Omidi N., Ghasemi S., Kashani I.R. (2019). Melatonin preconditioning of bone-marrow derived mesenchymal stem cells promotes their engraftment and improves renal regeneration in a rat model of chronic kidney disease. J. Mol. Hist..

[303] Zhao D., Yu Y., Shen Y., Liu Q., Zhao Z., Sharma R., Reiter R.J. (2019). Melatonin synthesis and function: Evolutionary history in animals and plants. Front. Endocrinol..

[304] Lin Y.W., Lee L.M., Lee W.J., Chu C.Y., Tan P., Yang Y.C., Chen W.Y., Yang S.F., Hsiao M., Chien M.H. (2016). Melatonin inhibits MMP-9 transactivation and renal cell carcinoma metastasis by suppressing Akt-MAPKs pathway and NF-DNA-binding activity. J. Pineal Res..

[305] Park E.J., Woo S.M., Min K.J., Kwon T.K. (2014). Transcriptional and post-transcriptional regulation of Bim controls apoptosis in melatonin-treated human renal cancer Caki cells. J. Pineal Res..

[306] Pourhanifeh M.H., Hosseinzadeh A., Juybari K.B., Mehrzadi S. (2020). Melatonin and urological cancers: A new therapeutic approach. Cancer Cell Int..

[307] Millet-Boureima C., Rozencwaig R., Polyak F., Gamberi C. (2020). Cyst reduction by melatonin in a novel *Drosophila* model of polycystic kidney disease. Molecules.

[308] Pei Y. (2001). A “two-hit” model of cystogenesis in autosomal dominant polycystic kidney disease?. Trends Mol. Med..

[309] Harris P.C. (2010). What is the role of somatic mutation in autosomal dominant polycystic kidney disease. J. Am. Soc. Nephrol..

[310] Eccles M.R., Stayner C.A. (2014). Polycystic kidney disease—Where gene dosage counts. F1000Prime Rep..

[311] Seeger-Nukpezah T., Geynisman D.M., Nikonova A.S., Benzing T., Golemis E.A. (2015). The hallmarks of cancer: Relevance to the pathogenesis of polycystic kidney disease. Nat. Rev. Nephrol..

[312] Sun K., Xu D., Mei C. (2019). The association between autosomal dominant polycystic kidney disease and cancer. Int. Urol. Nephrol..

[313] Shim K.E., Lee C., Kim J.U., Choi G.H., Kwak K.M., Kim S.H., Kim H., Yoon J.W., Shin T.Y., Jeong C.W. (2020). Comprehensive analysis of mutations of renal cell carcinoma in an autosomal dominant polycystic kidney disease patient. Medicine.

[314] Stark M.B. (1918). An hereditary tumor in the fruit fly, *Drosophila*. Cancer Res..

[315] Stark M.B. (1919). A benign tumor that is hereditary in *Drosophila*. Proc. Natl. Acad. Sci. USA.

[316] Stark M.B. (1919). An hereditary tumor. J. Exp. Zool..

[317] Wilson I.T. (1924). Two new hereditary tumors in *Drosophila*. Genetics.

[318] Herranz H., Eichenlaub T., Cohen S.M. (2016). Cancer in *Drosophila*: Imaginal discs as a model for epithelial tumor formation. Curr. Top. Dev. Biol..

[319] Pagliarini R.A., Xu T. (2003). A genetic screen in *Drosophila* for metastatic behavior. Science.

[320] Stuelten C.H., Parent C.A., Montell D.J. (2018). Cell motility in cancer invasion and metastasis: Insights from simple model organisms. Nat. Rev. Cancer.

[321] Tipping M., Perrimon N. (2014). *Drosophila* as a model for context-dependent tumorigenesis. J. Cell Physiol..

[322] Wu M., Pastor-Pareja J.C., Xu T. (2010). Interaction between Ras(V12) and scribbled clones induces tumour growth and invasion. Nature.

[323] Brumby A.M., Richardson H.E. (2003). scribble mutants cooperate with oncogenic Ras or Notch to cause neoplastic overgrowth in *Drosophila*. EMBO J..

[324] Grifoni D., Garoia F., Schimanski C.C., Schmitz G., Laurenti E., Galle P.R., Pession A., Cavicchi S., Strand D. (2004). The human protein Hugl-1 substitutes for *Drosophila* lethal giant larvae tumour suppressor function in vivo. Oncogene.

[325] Igaki T., Pagliarini R.A., Xu T. (2006). Loss of cell polarity drives tumor growth and invasion through JNK activation in *Drosophila*. Curr. Biol..

[326] Fahey-Lozano N., La Marca J.E., Portela M., Richardson H.E. (2019). *Drosophila* models of cell polarity and cell competition in tumourigenesis. Adv. Exp. Med. Biol..

[327] Eichenlaub T., Villadsen R., Freitas F.C.P., Andrejeva D., Aldana B.I., Nguyen H.T., Petersen O.W., Gorodkin J., Herranz H., Cohen S.M. (2018). Warburg effect metabolism drives neoplasia in a *Drosophila* genetic model of epithelial cancer. Curr. Biol..

[328] Kasai Y., Cagan R. (2010). *Drosophila* as a tool for personalized medicine: A primer. Per. Med..

[329] Sonoshita M., Cagan R.L. (2017). Modeling human cancers in *Drosophila*. Curr. Top. Dev. Biol..

[330] Bangi E. (2019). A *Drosophila* based cancer drug discovery framework. Adv. Exp. Med. Biol..

